# Long-Term Expansion in Platelet Lysate Increases Growth of Peripheral Blood-Derived Endothelial-Colony Forming Cells and Their Growth Factor-Induced Sprouting Capacity

**DOI:** 10.1371/journal.pone.0129935

**Published:** 2015-06-15

**Authors:** Dimitar Tasev, Michiel H. van Wijhe, Ester M. Weijers, Victor W. M. van Hinsbergh, Pieter Koolwijk

**Affiliations:** Dept. of Physiology, ICaR-VU, VU University Medical Center, Amsterdam, The Netherlands; IDI, Istituto Dermopatico dell'Immacolata, ITALY

## Abstract

**Introduction:**

Efficient implementation of peripheral blood-derived endothelial-colony cells (PB-ECFCs) as a therapeutical tool requires isolation and generation of a sufficient number of cells in *ex vivo* conditions devoid of animal-derived products. At present, little is known how the isolation and expansion procedure in xenogeneic-free conditions affects the therapeutical capacity of PB-ECFCs.

**Results:**

The findings presented in this study indicate that human platelet lysate (PL) as a serum substitute yields twice more colonies per mL blood compared to the conventional isolation with fetal bovine serum (FBS). Isolated ECFCs displayed a higher proliferative ability in PL supplemented medium than cells in FBS medium during 30 days expansion. The cells at 18 cumulative population doubling levels (CPDL) retained their proliferative capacity, showed higher sprouting ability in fibrin matrices upon stimulation with FGF-2 and VEGF-A than the cells at 6 CPDL, and displayed low β-galactosidase activity. The increased sprouting of PB-ECFCs at 18 CPDL was accompanied by an intrinsic activation of the uPA/uPAR fibrinolytic system. Induced deficiency of uPA (urokinase-type plasminogen activator) or uPAR (uPA receptor) by siRNA technology completely abolished the angiogenic ability of PB-ECFCs in fibrin matrices. During the serial expansion, the gene induction of the markers associated with inflammatory activation such as VCAM-1 and ICAM-1 did not occur or only to limited extent. While further propagation up to 31 CPDL proceeded at a comparable rate, a marked upregulation of inflammatory markers occurred in all donors accompanied by a further increase of uPA/uPAR gene induction. The observed induction of inflammatory genes at later stages of long-term propagation of PB-ECFCs underpins the necessity to determine the right time-point for harvesting of sufficient number of cells with preserved therapeutical potential.

**Conclusion:**

The presented isolation method and subsequent cell expansion in platelet lysate supplemented culture medium permits suitable large-scale propagation of PB-ECFC. For optimal use of PB-ECFCs in clinical settings, our data suggest that 15–20 CPDL is the most adequate maturation stage.

## Introduction

Prospective vascular regenerative therapies rely on suitable sources of endothelial cells that can be propagated in *ex vivo* conditions devoid of the presence of animal-derived products. Isolation and generation of sufficient numbers of endothelial cells required for delivery into patients demands identification of defined cell populations which growth and function can be monitored in xenogeneic-free cell culture conditions. Limited proliferative and vasculogenic potential of mature endothelial cells such as human umbilical vein endothelial cells (HUVEC)[1–2] and human dermal microvascular endothelial cells (HMEC)[3–4] restrain the use of endothelial cells derived from autologous tissue for future tissue engineering and cell-based therapies. A specific subgroup of circulating endothelial progenitor cells (EPCs), present in cord and adult peripheral blood, represent a promising source for in vitro expansion and obtaining sufficient endothelial cells for clinical application.

Recent advances in the field of vascular biology have identified two distinct types of EPCs, depending on the time of appearance during isolation period. Early outgrowth EPCs, which are derived from the myelo-monocytic lineage, appear in culture during the first seven days of isolation and participate in the processes of vascular regeneration in a paracrine fashion[5]. On the contrary, the late outgrowth EPCs also known as endothelial-colony forming cells (ECFCs) appear in the cell culture usually after 10 days of isolation and are not derived from myelo-monocytic lineage. The peripheral and cord blood-derived ECFCs, characterized by robust proliferative capacity[6] and vessel forming ability in vivo[7], are currently considered as a viable cell source for future clinical application, both as a source for tissue repair and to study pathophysiological mechanisms at cellular level in patients.

These cells originate from circulating stem/progenitor cells which are easily encountered in cord blood, yet exceedingly rare in peripheral blood[8].

At present, efficient isolation of ECFCs relies on creating an *in vitro* environment that favors differentiation of the progenitor cell into an endothelial lineage. Current protocols for isolation and expansion of peripheral blood-derived ECFCs (PB-ECFCs) depend mostly on supplementing the cell culture medium with 10–20% (v/v) of FBS. However, the transfer of potentially harmful xenogeneic material[9] and the induction of an immune response[10–11] in human recipients to FBS restricts the use of animal-derived products for isolation and *ex vivo* expansion of PB-ECFCs. Therefore, alternative xenogeneic-free approaches for isolation and *ex vivo* manipulation of PB-ECFCs prior to clinical application are greatly needed. Previous experience with *ex vivo* expansion of mesenchymal stem cells (MSCs) suggests that human blood-derived products such as platelet lysate (PL)[12], serum[13], and platelet-rich plasma (PRP)[14] are feasible FBS substitutes.

Successful application of ECFCs from adult blood as a therapeutic tool relies on prolonged expansion in culture conditions that might have impact on cell physiology. Serial passaging during expansion affects the response of cells to the environmental factors. Previous studies have identified that long-term expansion reduces the therapeutical efficiency of hematopoietic stem cells (HSC)[15]. ECFCs expanded in medium supplemented with FBS are prone to high incidence of karyotype changes[16] and exhibit a decreased angiogenic ability[7,17]. On the contrary, long-term expansion of ECFCs in medium supplemented with a FBS alternative, in particular platelet lysate, preserved genomic stability of the cells[18]. However, we had to modify this procedure since we and others[19] were not able to isolate ECFCs from peripheral blood directly. Furthermore, little is known whether the expansion procedure in animal-free conditions affects the angiogenic ability of PB-ECFCs.

In this study, we compare the efficiency of initiation of PB-ECFCs cultures in xenogeneic-free settings based on use of platelet lysate as serum supplement with the current widespread used procedure which relies on FBS. In addition, we investigate whether the angiogenic ability of PB-ECFCs is affected by long-term expansion in medium supplemented with platelet lysate. Finally, the underlying mechanism that governs the sprout formation in fibrin matrices was studied. The findings presented in this study indicate that 1) platelet lysate is a better culture medium supplement than FBS for isolation and *ex vivo* propagation of PB-ECFCs, 2) cells expanded in platelet lysate retain their endothelial phenotype, 3) extensive propagation increases the angiogenic capacity of PB-ECFCs, and 4) sprout formation in fibrin matrices depends on PAI-1 (plasminogen-activator inhibitor) balanced activity of the uPA/uPAR system.

## Materials and Methods

### Preparation of pooled human platelet lysate

Platelet-rich plasma (PRP) for platelet lysate was prepared by the blood transfusion service (Department of Hematology, VU University Medical Center, Amsterdam, The Netherlands) accordingly the protocol of Korte et al[20]. Upon arrival, the bags of 5 whole blood donations, each containing 10^9^ platelets per mL was stored at -80°C. A batch of pooled PL consisting whole blood donations obtained from 35 healthy donors were prepared after thawing the bags of PRP followed by centrifugation at 4075x g for 15 minutes to remove the residual platelet fragments. The platelet lysate for cell culture was aliquoted and stored at -20°C. Prior to preparation of cell culture medium, the PL was thawed and centrifugated again at 4075 x g for 15 minutes. Similar results were obtained with a second pooled PL preparation based on blood of 70 healthy donors.

### Isolation and culture of ECFCs from adult peripheral blood

Peripheral blood samples (PB) were collected from 10 healthy donors (5 males and 5 females, age range 21–50 years, average age 30±8SD years) after obtaining written informal consent in accordance with the institutional guidelines. To isolate mononuclear cell (MNCs) fraction, the blood was pre-diluted 1:1 with pre-warmed phosphate buffered saline solution(PBS) and transferred into LeucoSept tubes (Greiner Bio-one, The Netherlands) previously filled with FicolPaque Plus (GE Healthcare Europe GmbH, The Netherlands). After centrifugation at 740 x g for 30 min, the interphase with MNC was collected and washed three time with M199 (Lonza, Verviers, Belgium) supplemented 0.1% penicillin/streptomycin (Invitrogen, The Netherlands). After final washing, the MNC were re-suspended either in complete EGM-2 (Lonza, Walkersville, MD, USA) supplemented with 10%FBS (FBS-EGM) or with 10% platelet lysate (PL-EGM), 0,1% penicillin-streptomycin, 2 mM L-glutamine and 10 U/mL heparin, and seeded at density of 1.3*10^6^cells/cm^2^ (~1mL blood per one well) into 48 well-plates pre-coated with 3μg/cm^2^ human collagen type I prepared according to manufacturer’s instructions(#C7624, Sigma Aldrich). After 24 hours, nonadherent cells were carefully removed and fresh medium was added to each well. Medium was changed daily for 7 days and then every 2 days. After 10–30 days distinct ECFC colonies with regular cobblestone morphology appeared in the cell culture. The outgrowth ECFC colonies were enumerated by daily visual inspection using phase-contrast microscopy. Primary clones of PB-ECFCs were detached using Accutase (#A6964, Sigma Aldrich) and cells re-plated onto one well of a 12 well plate were designated as passage 0. For further expansion and experimental purposes cells were detached with 0.05% trypsin/EDTA and seeded at density of 5000 cells/cm^2^onto cell-culture plates pre-coated with 5μg/cm^2^ rat-tail collagen type I (BD Biosciences, Erebodegem, Belgium) for each passaging step in PL-EGM or FBS-EGM.

### Immunophenotyping of PB-ECFCs and uptake of Dil-Ac-LDL

Isolated cells were characterized for uptake of Ac-Dil-LDL (Biomedical Technologies Inc, USA) according to the manufacturer's protocol. Expression of endothelial lineage surface markers was evaluated by flow cytometry (FACS Calibur; BD Biosciences) at 18 CPDL using PE mouse anti-human CD34 (IgG1, 555822), PE mouse anti-human CD144 (IgG1, 561714), PE mouse anti-human CD31 (IgG1, 555446), PE mouse anti-human CD309 (IgG1, 560872), all purchased from BD Biosciences, as well as FITC mouse anti-human CD146 (IgG1, 130-092-851, MiltenyiBiotec) and PE mouse anti-human CD105 (IgG1, MHCD10504, Invitrogen). Flow cytometry detection of hematopoietic cells was performed using antibodies against hematopoietic cell–specific surface antigens such as PE mouse anti-human CD133/1 (AC133) (IgG1, 130-080-801, MiltenyiBiotec), FITC Mouse Anti-Human CD45 (IgG1, 555748, BD Bioscience), FITC mouse anti-human CD14 (IgG2a, 555397, BD Bioscience). Appropriate isotype controls PE mouse anti-human IgG1 isotype (IgG1, 555749), FITC mouse anti-human IgG1 isotype (IgG1, 555748), and PE mouse anti-human IgG2a (IgG2a, 559319) were purchased from BD Bioscience. Data were analyzed by using the FCS Express 4 software package (De Novo Software, Toronto, Ontario, Canada).

For immunocytochemistry phenotyping, cells were seeded on gelatin coated glass cover slips, fixed with 4% paraformaldehyde/EDTA and permeabilized with 0,5% Triton X-100/PBS. After washing, cells were incubated with unconjugated primary antibodies (goat anti-VE-Cadherin clone C-19, rabbit anti-vWF clone H-300, and goat anti-PECAM-1 clone M-20, all from Santa Cruz Biotechnology, USA) prepared in PBS containing 0,1% human serum albumin (HSA) for 24 h at 4°C. The next day, after two washing steps, cells were incubated with appropriate FITC labeled secondary antibodies (rabbit anti-goat IgG or donkey anti-rabbit IgG Alexa 488, Molecular probes, Invitrogen, USA) prepared in 0,1% HSA for 2 h in dark room. Cell nuclei were visualized with DAPI VectashieldHardSet present in the mounting medium (VectorLabLtd, BrunschwigChemie, The Netherlands) prior to immunofluorescence examination using 4D-digital imaging microscope (DIM).

### Growth kinetics of PB-ECFCs and proliferation assay

To determine the proliferative capacity of PB-ECFCs in PL-EGM or FBS-EGM, viable cells were enumerated at passage 1 using hemocytometer and trypan blue exclusion assay. For each subsequent passage, 5000 cells/cm^2^ were seeded on 6-well plates pre-coated with rat-tail collagen type I. The cell number after each passaging step was used to calculate the total number of yielded cells, the CPDL, and the population doubling times (PDT) as previously reported[6]. CPDL values were calculated during this propagation period (40–42 days) and did not include the cell proliferation during initial clonal outgrowth.

For short-term proliferation assays 500 cells/cm^2^ were seeded onto 12-well plate pre-coated with rat-tail collagen type I in PL-EGM or FBS-EGM. Renewal of culture medium was performed every other day. After 7 days in culture, the cells were washed with PBS and fixed with pre-warmth 2% paraformaldehyde/HBSS. Crystal violet staining was used to visualize the cell nuclei and 5 pictures from each well were taken using phase contrast microscopy. The number of cells was determined using ImageJ software.

### Tube-formation fibrin assay

Assessment of sprouting ability of PB-ECFCs expanded in PL-EGM was performed at 6, 18, and 31 CPDL by seeding 20.000 cells on 3D human fibrin matrices prepared as previously described[21]. Following overnight incubation in M199 supplemented with 10% inactivated human serum and 10% new-born calf serum, tube formation was induced by stimulating the cells with either 10ng/ml TNF-α (T), 10ng/ml FGF-2 (F) or 25ng/ml VEGF_165_(V) alone, or the combinations of them (TF: TNF-α+FGF-2, TV: TNF-α+ VEGF_165_, TFV: TNF-α+FGF-2+VEGF_165_, FV: FGF-2+VEGF_165_). All growth factors were purchased from ReliaTech GmbH, Wolfenbuttel, Germany. To investigate the effect of FBS and PL on tube-formation of PB-ECFCs in fibrin matrices, the cells were were stimulated twice with 25ng/mL VEGF-A prepared in M199+10%FBS+10U/mL heparin or M199 +5%PL+10U/mL heparin. After 48h stimulation, the cells were fixed with 2% paraformaldehyde/HBSS and quantification of the length of formed tube-like structures was performed using Optimas image analysis software as previously described[21]. The tube formation ability of PB-ECFCs of 3 donors was determined in triplicate wells.

### siRNA transfection tube-formation assay

To investigate the involvement of uPA, uPAR and PAI-1 in sprout formation in fibrin matrices by PB-ECFCs expanded in PL, siRNAs against uPA (Hs_PLAU_6 FlexiTube siRNA, cat.no. SI02662135) or uPAR (Hs_PLAUR_6 FlexiTube siRNA, cat.no. SI03048458) were purchased from QiagenBenelux B.V., the Netherlands and prepared according to manufacturer’s instructions. Pool of target-specific siRNAs against PAI-1 (sc-36179) was purchased from Santa Cruz Biotechnologies, USA. ON-TARGETplus Non-targeting Pool siRNA (cat.no. D-001810-10-05) was purchased from GE Dharmacon, Lafayette, CO. Prior transfection experiments cells were starved for 4h in M199 and were transfected using DharmaFECT4 reagent (Dharmacon). All siRNA and DharmaFECT4 were prepared in M199 + 10% inactivated human serum supplemented with 10ng/mL FGF-2 at final concentration of 20nM. The transfection medium was replaced by fresh standard PL-EGM medium, 24h post-transfection. Transfection efficiency was evaluated by qRT-PCR after additional 24h period of recovery. At the same time point, the cells were seeded onto fibrin matrices and sprout formation was initiated by stimulating the cells with combination of 10ng/mL TNF-a and 10ng/mL FGF-2 every day during 3-days period in medium as already described in the previous section. Inhibition of tube formation was accomplished by preventing the conversion of plasminogen to plasmin by adding 100U/mL aprotinin (100U/mL) to the stimulation medium consisting TNF-a and FGF-2. The tube formation ability of PB-ECFCs of 4 donors was determined in triplicate wells. Quantification of the length of formed tube-like structures was performed as already described in the previous section.

### ELISA of uPA and PAI-1

For ELISA determination of soluble uPA antigen in conditioned medium, the PB-ECFCs of three donors at 6, 18, and 31 CPDL were previously starved in EBM-2 + 5%PL for 24h. After starvation period the conditioned medium was collected and centrifuged to remove the cell debris. Collected supernatant was used to determine concentration of soluble uPA as previously described[22]. The concentration of soluble human Serpin E-1 (PAI-1, cat.num.DY1786, R&D systems, Minneapolis, MN) in conditioned medium was determined by ELISA following manufacturer's instructions.

### Real-Time Polymerase Chain Reaction

For RNA isolation the PB-ECFCs of three donors at 6, 18, and 31 CPDL prior to collection of cell lysates were previously starved in EBM-2 + 5%PL for 24h. The total RNA was isolated using RNeasyMinElute Cleanup Kit (Qiagen, The Netherlands) and the RNA quality was tested with a Nanodrop 1000 spectrophotometer. Copy DNA (cDNA) was synthesized using the Cloned AMV First Strand cDNA Synthesis Kit from Invitrogen. The sequences of primers used for determination of genes of interest are given in **S1 Table**. Quantitative RT-PCR was performed using SYBR Green in an ABI 7500 sequence detection system (Applied Biosystems, Foster City, USA) and the following protocol: 2 min 50°C, 10 min 95°C and 40 cycles (15 sec 95°C, 1 min 60°C) and dissociation curve. The relative expression levels of target genes were calculated with the housekeeping gene glyceraldehyde 3-phosphate dehydrogenase (GAPDH) by following equation as previous described[23]: Δ Ct sample = (Ct sample GENE)–(Ct sample HKG). The relative gene expression = 2 ^(Δ Ct sample1 –Δ Ct Sample)^.

### Determination of telomere length

The telomere length of PB-ECFCs from three different donors at 6, 18, and 31 CPDL was determined by quantitative real-time polymerase chain reaction (QPCR) as previously described[24]. DNA was isolated using DNeasy kit (Qiagen) following the manufacture's protocol. The sequences of the primers for single-copy gene (*36B4*) and telomeres are specified in literature[24]. QPCR was performed in duplicate wells using SYBR Green in an ABI 7500 sequence detection system (Applied Biosystems, Foster City, USA). The relative telomere length was determined using the telomere/single gene (T/S) ratio with the calculation [2^Ct (telomeres)^/2^Ct (single-copy gene)^] = 2^-ΔCt^.

### Senescence-Associated β-Galactosidase Staining

Senescence-associated β-galactosidase (SA-β-Gal) activity in PB-ECFCs was evaluated with a Cellular senescence assay (#KAA002, Merck Milipore) following the manufacturer's protocol.

### Statistical analysis

Data are expressed as means ± SEM. At least three independent experiments were performed for all analyses. Single comparisons were made with Student's t tests for normally distributed data or the Wilcoxon matched-pairs signed rank test for data not normally distributed. Significance was defined as a p value of <0.05. Comparisons between multiple groups were performed using one- or two-way ANOVA with Bonferroni post hoc test.

## Results

### Isolation of PB-ECFCs in medium supplemented with platelet lysate

**Fig 1:**
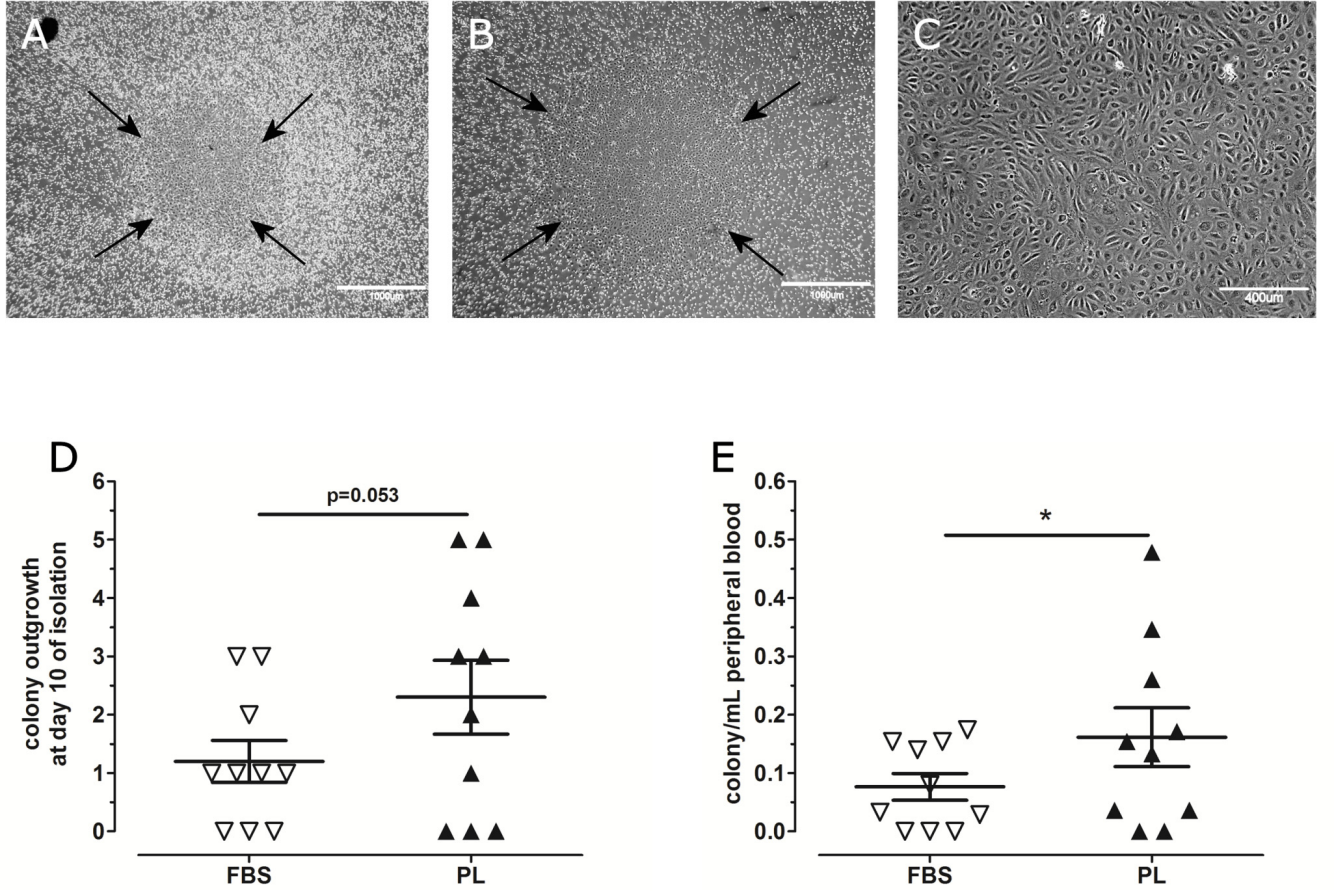
Comparison of ECFCs colony outgrowth in fetal bovine serum and platelet lysate. Representative images of ECFC colonies that appeared in FBS-EGM (**A**) and PL-EGM (**B,C**) between 10–30 days. (**D**): The average number of colonies that grew in FBS (open triangle) and PL (black triangle) from 10 isolations at day 10. (**E**): The number of colonies yields in FBS-EGM-2 (open triangle) or PL-EGM (black triangle) during the isolation period in all donors relative to the number of mL of peripheral blood. Data are expressed as mean±SEM. Statistical analysis was performed with non-parametric Wilcoxon matched pairs test (* p<0.05).

Mononuclear cells collected from adult peripheral blood of 10 individual donors (5 males, 5 females) were divided and inoculated on human collagen type-I pre-coated plates in EGM-2 medium supplemented either with 10% FBS or 10%PL. After 10 days, both culture conditions gave rise to the appearance of colonies with a cobblestone morphology characteristic for ECFCs (**Fig 1A**, FBS and **Fig 1B and 1C**, PL). During the first 10 days of initial plating, the MNCs in EGM-2 medium supplemented with 10%PL generated more colonies per total volume of collected blood than MNC in EGM-2 with 10%FBS (2.3 vs. 1.2 colonies in PL-EGM and FBS-EGM, respectively; p = 0.053; **Fig 1D**). At the end of the isolation period of 30 days, two times more colonies per mL of PB were counted in the cultures initiated with PL-EGM than the cultures plated in FBS-EGM (0.16 vs.0.08 colonies/mL PB, p = 0.04; **Fig 1E**; or 0.11 colonies/10^6^MNC vs. 0.06 colonies/10^6^MNC). This indicates that PL is a more efficient serum supplement for isolation of PB-ECFCs than the current standard protocol based on the use of FBS.

### Phenotypical characterization of PB-ECFCs isolated in platelet lysate-containing medium

**Fig 2:**
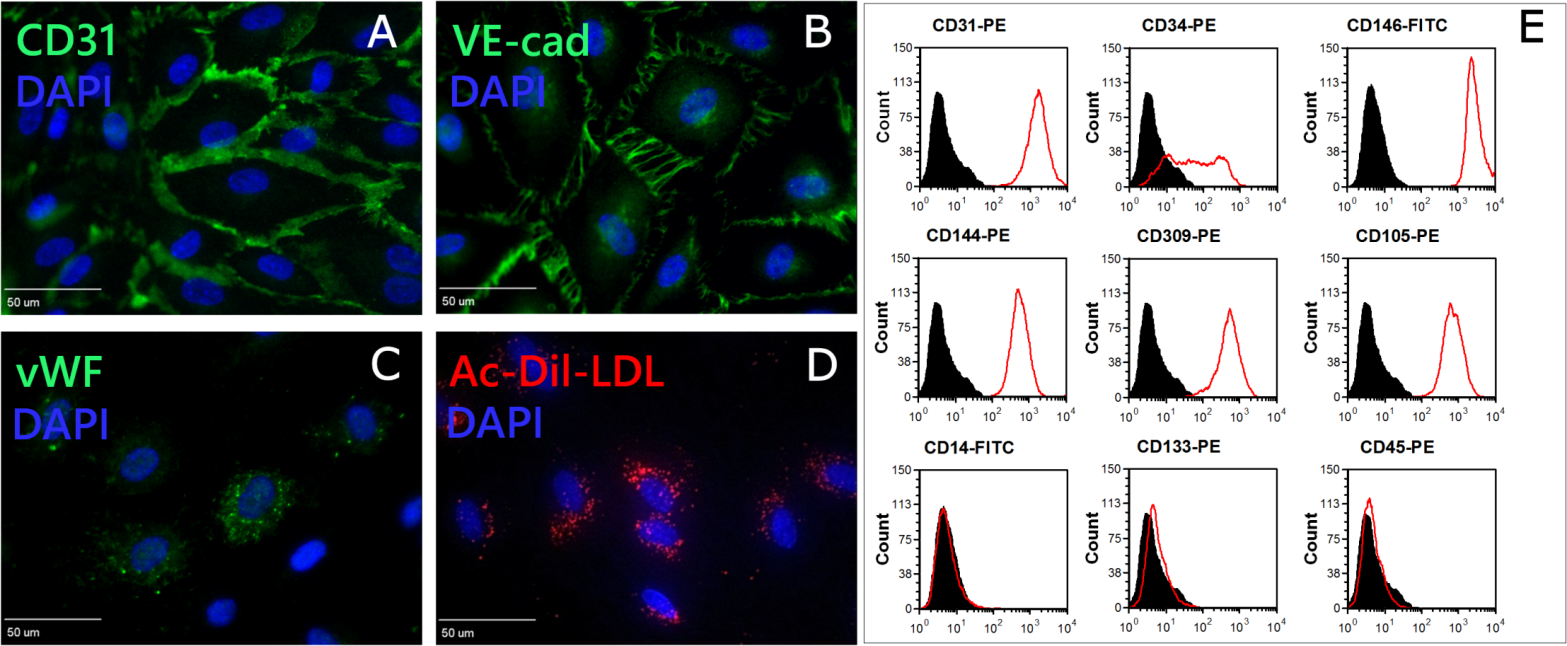
Phenotypical characterization of PB-ECFCs. PB-ECFCs monolayers expanded in EGM-2 medium supplemented with 10%PL were assessed for the presence of endothelial cell markers by immunofluorescence cytochemistry (**A-C**) and flow cytometry as well as for uptake of Dil-Ac-LDL (**D**). For immunofluorescence cytochemistry staining, cells were seeded on glass cover slips, fixed and stained with antibody against CD31, VE-cadherin or vWF. Cell nuclei were visualized with DAPI staining. Cells were positive for CD31 (**A**, green), VE-cadherin (**B**, green), and vWF (**C**, green). Cell nuclei appear blue. (**D**): Incorporation of Dil-Ac-LDL by PB-ECFCs (red spots, cell nuclei stained blue with DAPI). Panel **E**:Flow cytometry characterization of PB-ECFCs for CD31, CD34, CD309, CD144, CD146, CD105, CD14, CD45, and CD133. Plots depict control isotype IgG staining (black histograms) versus specific antibody staining (empty histograms).

To determine the endothelial phenotype of PB-ECFCs isolated in PL-EGM, the primary colonies from the donors were serially expanded by seeding 5000 cells/cm^2^ in PL-EGM. Immunofluorescence cytochemisty confirmed the expression of endothelial marker CD31, CD144, vWF in the PB-ECFCs as well as the uptake of Ac-Dil-LDL (**Fig 2A–2D**). The flow cytometric immunophenotyping with antibodies specific for endothelial cell lineage markers confirmed the endothelial nature of PB-ECFCs (**Fig 2E**). The cells were positive for CD31, CD34, CD144, CD146, CD309, CD105 and negative for hematopoietic cell surface antigens CD14, CD45, and CD133 which is in accordance with previously published data[6].Altogether, these data show that PB-ECFCs isolated and expanded in platelet lysate resembles the immunophenotype of the ECFCs isolated and expanded in FBS[6].

### Increased proliferation of PB-ECFCs by platelet lysate

**Fig 3:**
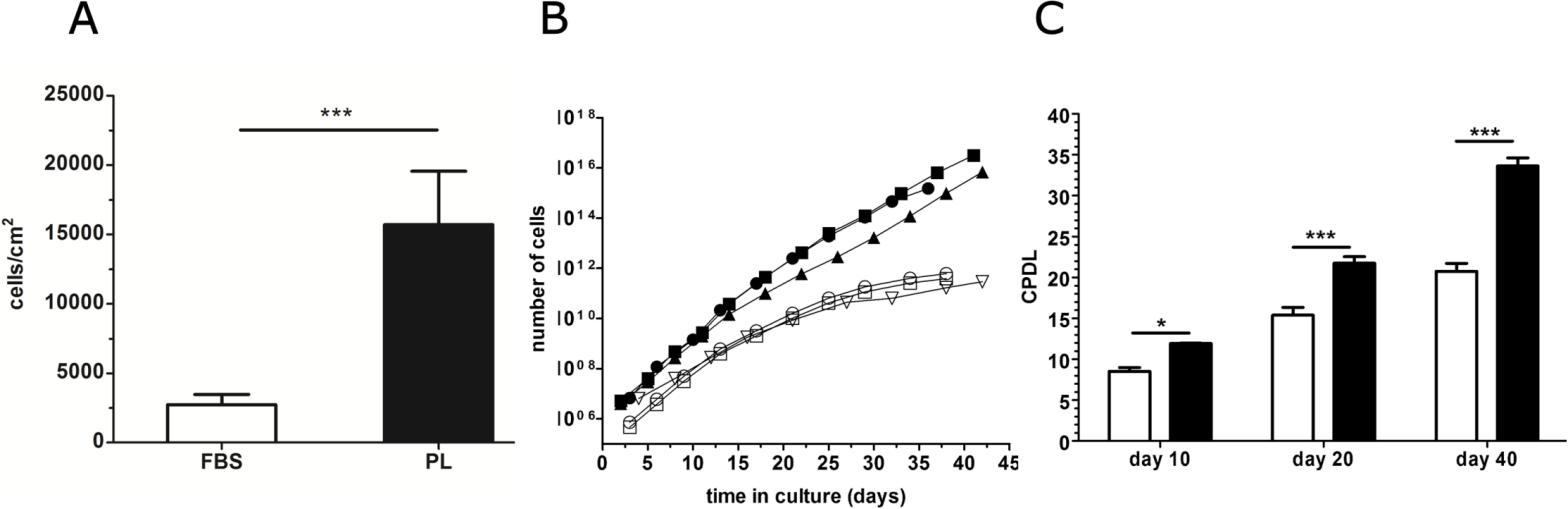
Proliferative potential of PB-ECFCs in FBS and PL medium. For growth kinetics as well as cumulative population doubling determination experiments, cells were serially expanded by seeding 5000 cells/cm^2^ in EGM-2 supplemented with either 10%FBS or 10%PL during period of 40 days. (**A**): Comparison of proliferative potential of PB-ECFCs maintained in FBS-EGM (open bar) or PL-EGM (closed bar) during 7 days. Results represent the mean ± SEM of counted number of cells relative to cm^2^ of plating surface of 3 independent experiments of three different donors. *p < 0.05 by Student paired t test. (**B**): Total number of cells yielded during long-term expansion of PB-ECFCs in FBS-EGM (open symbol, n:3) or PL-EGM (closed symbols, n:3). Each symbol indicates total number of cells at each passaging step. (**C**): Cumulative population doubling levels (CPDL) of PB-ECFCs in PL-EGM (closed bars) or FBS-EGM (open bars) after 10, 20, and 40 days of expansion. Results represent the mean ± SEM of CPDL at three different time points of 3 independent experiments of 3 different donors. Two-way ANOVA with Bonferroni post hoc test.

The proliferative ability of PB-ECFCs during the short-term (7 days) growth was significantly faster rate in PL-EGM than in FBS-EGM (**Fig 3A**). Subsequently, the growth kinetics of PB-ECFCs from three donors were evaluated simultaneously in FBS or PL during the same period of 40 days. PB-ECFCs serially expanded in FBS generated less cells (4.11*10^11^cells vs. PL: 2.9*10^15^cells, **Fig 3B**) and showed lower CPDL (Day 40: CPDL_FBS_ 20.7 vs. CPDL_PL_ 33.6, **Fig 3C**) than their counterparts expanded in PL. These results show that PL is more suitable as a serum supplement than FBS, not only for isolation, but also for long-term expansion of PB-ECFCs.

### Loss of progenitor status reduces proliferation of PB-ECFCs during ex vivo expansion

**Fig 4:**
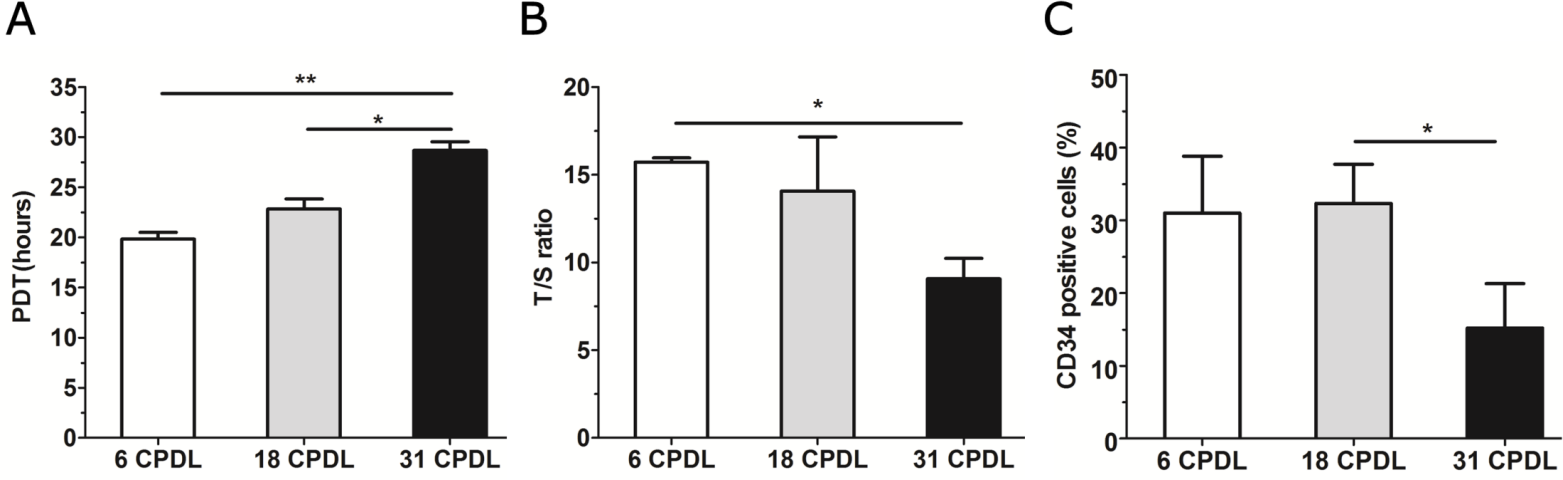
Comparison of proliferation-related features in PB-ECFCs during long-term cell expansion. During long-term expansion of PB-ECFCs in PL-EGM, a set of experiments at different CPDLs (white bar 6CPDL, grey bar 18CPDL, black bar 31CPDL) were performed to investigate proliferation rate, telomere length and expression of the progenitor-cell marker CD34. (**A)** Comparison of population doubling time (PDT). (**B**): Telomere (T) and single copy gene (S) were amplified by quantitative real-time PCR, with the T/S ratio proportional to telomere length at different stages of cells’ age. (**C**): Flow cytometry data of percentage of cells positive for CD34. Results represent the mean ± SEM of 3 independent experiments each performed with different donor at indicated CPDL. Comparison between each CPDL was performed by one-way ANOVA with Bonferroni post hoc test (*p<0.05).

The ability to maintain telomere length during consecutive cell divisions as well as the high expression of the progenitor-related CD34 antigen on the cell surface are two hallmarks of progenitor cells. We subsequently evaluated whether these parameters become altered during serial propagation of PB-ECFCs at 6CPDL (7 days of culture), 18CPDL (19 days), and at 31CPDL (38 days) in PL-EGM containing medium. PB-ECFCs can serially be expanded in PL-EGM for more than 30 CPDL within 40 days with an average population doubling time (PDT) of 23.8±1.0 h per cell cycle. Comparison of the PDT at different time intervals during the culture period showed that the cells at 31 CPDL exhibit significant longer PDT (28.6±1.5 h) than the cells at 6 CPDL (19.8±1.1 h) or 18 CPDL (22.8±1.7 h) respectively, indicating a decline in proliferation rate (**Fig 4A**).

The reduction in proliferation was reflected in the dynamics of telomere shortening. A significant telomere shortening was detectable only in the cells at 31 CPDL (**Fig 4B**). To investigate if this decrease was caused by an accumulation of senescent cells, β-galactosidase staining was performed. Although at 6, 18 and 31 CPDL a few β-galactosidase positive cells were detectable, there was no difference in their numbers between these conditions (data not shown).

In addition, we investigated if the observed reduction in the rate of cell division at 31 CPDL might be reflected in a decline of CD34^+^ cells in the culture. Flow cytometric analysis revealed a gradual decrease in the percentage of CD34^+^ ECFCs during the 40 days of culture; 31.0%, 32.3% and 15.2% CD34^+^cells at 6, 18 and 31 CPDL, respectively (**Fig 4C**). Additional analysis of mean fluorescence intensity by flow cytometry and gene expression of CD34 by QPCR confirmed the decline of expression of this antigen at protein and gene level in time (**S2 Fig**). These data indicate that the moderate attenuation of proliferation of PB-ECFCs at 31 CPDL is associated with detectable telomere shortening and reduced numbers of CD34^+^ cells during *in vitro* expansion, which reflects a gradual loss of progenitor hallmarks during serial propagation.

### Effect of long-term expansion on the sprouting response of PB-ECFCs

**Fig 5:**
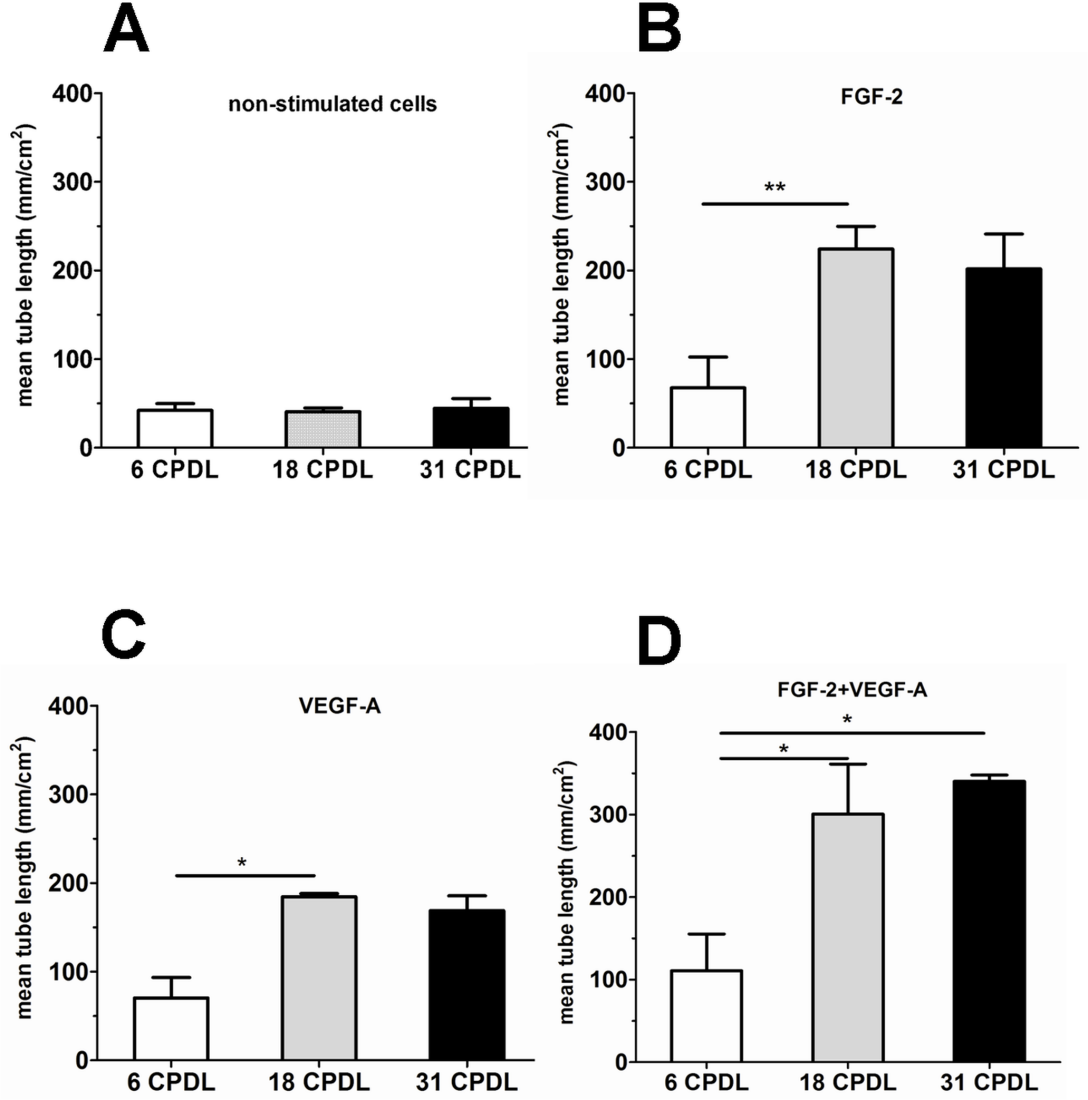
Comparison of tube-forming capacity of PB-ECFCs at different time-points of ex vivo expansion upon stimulation with VEGF-A and FGF-2. PB-ECFCs obtained from 3 different donors were serially expanded in medium supplemented with PL and the sprouting ability of cells in fibrin matrices was assessed at 6, 18, and 31 CPDL. Cells at indicated CPDL (white bar 6 CPDL, grey bar 18 CPDL, black bar 31 CPDL) were either unstimulated (**A**) or stimulated with FGF-2(**B**), and VEGF-A(**C**), FGF-2+VEGF-A (**D**),TNF-α. Results represent the mean ± SEM of mean tube length of tube-like structures of the 3 donors each performed at indicated CPDL. Comparison between each CPDLs was performed using one-way ANOVA with Bonferroni post hoc test.(*p < 0.05).

Fibrin is the most abundant protein present in the provisional extracellular matrix during angiogenesis. Therefore, we evaluated whether long-term expansion affects the sprouting capacity of PB-ECFCs in 3D fibrin matrices at different CPDL (6, 18, and 31 in PL-EGM). Quantification of the sprouting capacity of PB-ECFCs at 6, 18, and 31 CDPL (**Fig 5B and 5C**) showed that cells at 18 CPDL responded much stronger to the sole addition of FGF-2 or VEGF-A than their counterparts at 6 CPDL. Combined addition of FGF-2 and VEGF-A induced more sprout formation in cells at 18 and 31 CPDL than in 6 CPDL cells (**Fig 5D**). TNF-α also stimulated sprouting (Fig A in **S3 Fig**). Interestingly, addition of TNF-α to FGF-2 or VEGF-A or FGF-2+VEGF-A completely abolished the difference in angiogenic sprouting between cells at 6 CPDL and 18 or 31 CPDL that was observed when sole growth factors were used (Figs B-D in S3 **Fig**).

In aggregate, the data indicate that PB-ECFCs give a direct sprouting response upon exposure to TNF-α, and that 18 and 31 CPDL PB-ECFCs respond more avidly to single angiogenic growth factors than 6 CPDL cells.

### Increased angiogenic response of PB-ECFCs in fibrin matrices is accompanied by intrinsic upregulation of the fibrinolytic system

**Fig 6:**
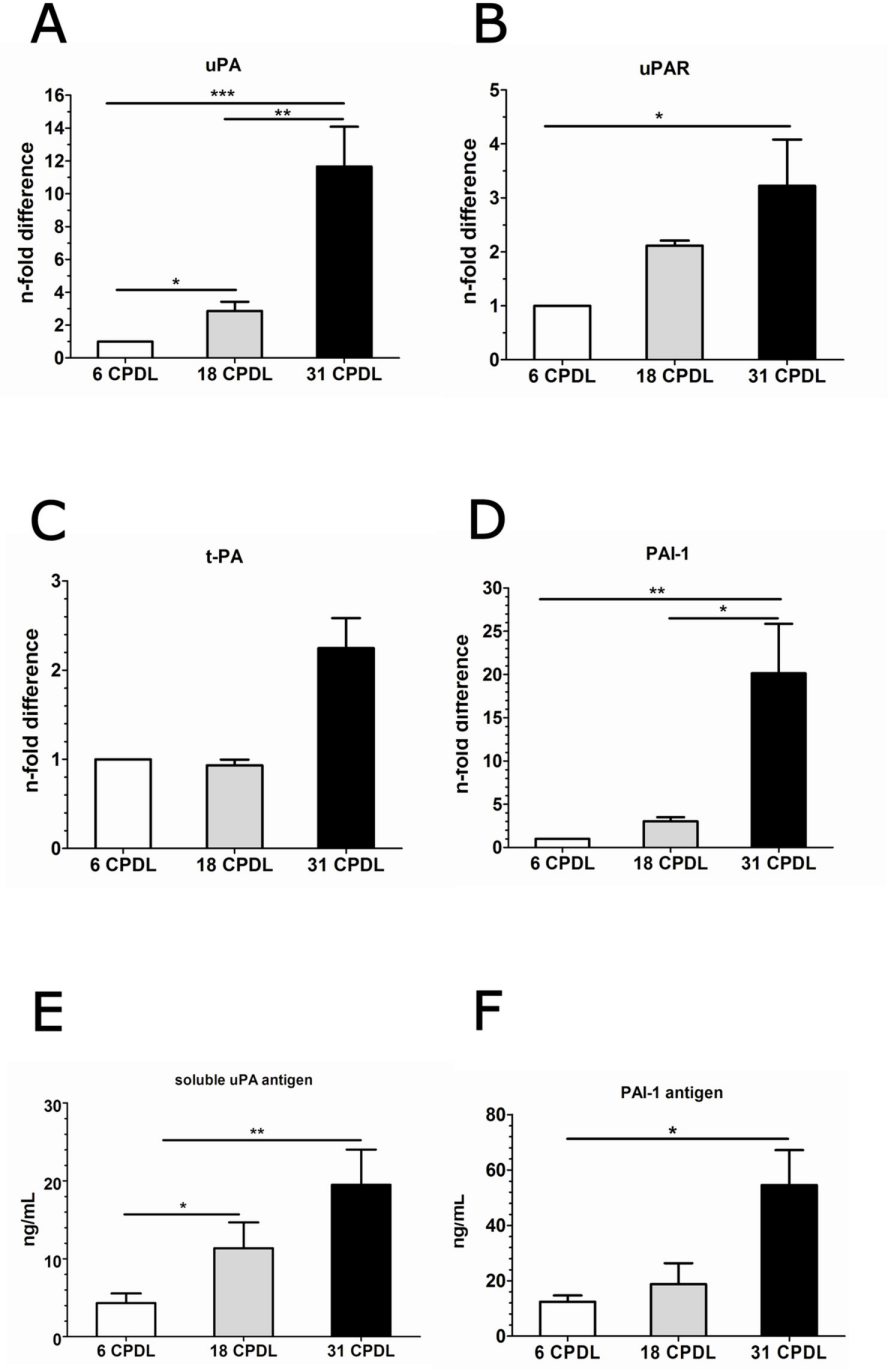
Expression of genes and soluble antigens involved during sprouting in fibrin matrix in PB-ECFCs at different maturation stages. Quantitative RT-PCR analysis was performed on total cellular mRNA isolated from PB-ECFCs at different CPDL (open bar 6 CPDL, grey bar 18 CPDL, black bar 31 CPDL). Gene expression levels of uPA (**A**), uPAR (**B**), tPA (**C**), and PAI-1 (**D**) in PB-ECFCs. Data are expressed as n-fold difference of expression of genes in cells at 6 CPDL. One-way ANOVA with Bonferroni post hoc test (p<0.05).Evaluation of the production of uPA (**E**) and PAI-1 (**F**) antigens in CM by PB-ECFCs was performed at 6 CPDL (open bar), 18 CPDL (grey bar), and 31 CPDL (black bar) using ELISA. Results represent the mean ± SEM of uPA or PAI-1 concentration in ng relative to mL of conditioned medium of 3 independent experiments each performed at indicated CPDL. Comparison between different groups was performed one-way ANOVA with Bonferroni post hoc test (p<0.05).

A finely tuned degradation of the fibrin matrix via the PAI-1-balanced activity of uPA/uPAR/plasmin system at the surface of endothelial cells ensures proportional fibrinolysis and adequate angiogenesis. We therefore evaluated the mRNA expression of uPA, uPAR, tPA (tissue-tyoe plasminogen activator) and PAI-1 in ECFCs at 6, 18, and 31 CPDL. The basal expression of uPA, uPAR and PAI-1 genes was considerably upregulated in PB-ECFCs at 18 and 31 CPDL compared to the cells at 6 CPDL (**Fig 6A, 6B and 6D**).

To evaluate whether the increased mRNA levels of uPA and PAI-1 induce proportional protein synthesis we assessed the amount of these two soluble antigens in conditioned medium (CM) collected at various CPDL. As illustrated in **Fig 6E**, soluble uPA antigen was prominently present in CM of PB-ECFCs at 18 CPDL and 31 CPDL. With respect to the amount of released soluble PAI-1 in CM, cells at 31 CPLD produced significantly more PAI-1 only in comparison to 6 CPDL cells but not to the cells at 18 CPDL (**Fig 6F**). In addition, cells at 6 and 18 CPLD produced comparable amount of soluble PAI-1 in CM under basal, unstimulated conditions. MMP-1, MMP-2 and MMP-14 were also clearly expressed in PB-ECFCs (Ct values at 6 CPDL 16.2±0.4, 20.0±0.1 and 22.1±1.3 mean±SEM, 3 donors), while MMP-9 expression was very low (Ct values at 6 CPDL 30.8±0.4mean ± SEM, 3 donors). There was no statistical difference or general trend of the changes of these MMPs during serial propagation (data not shown).

### Activation of uPA/uPAR/PAI-1 system directs sprout formation in fibrin matrices

**Fig 7:**
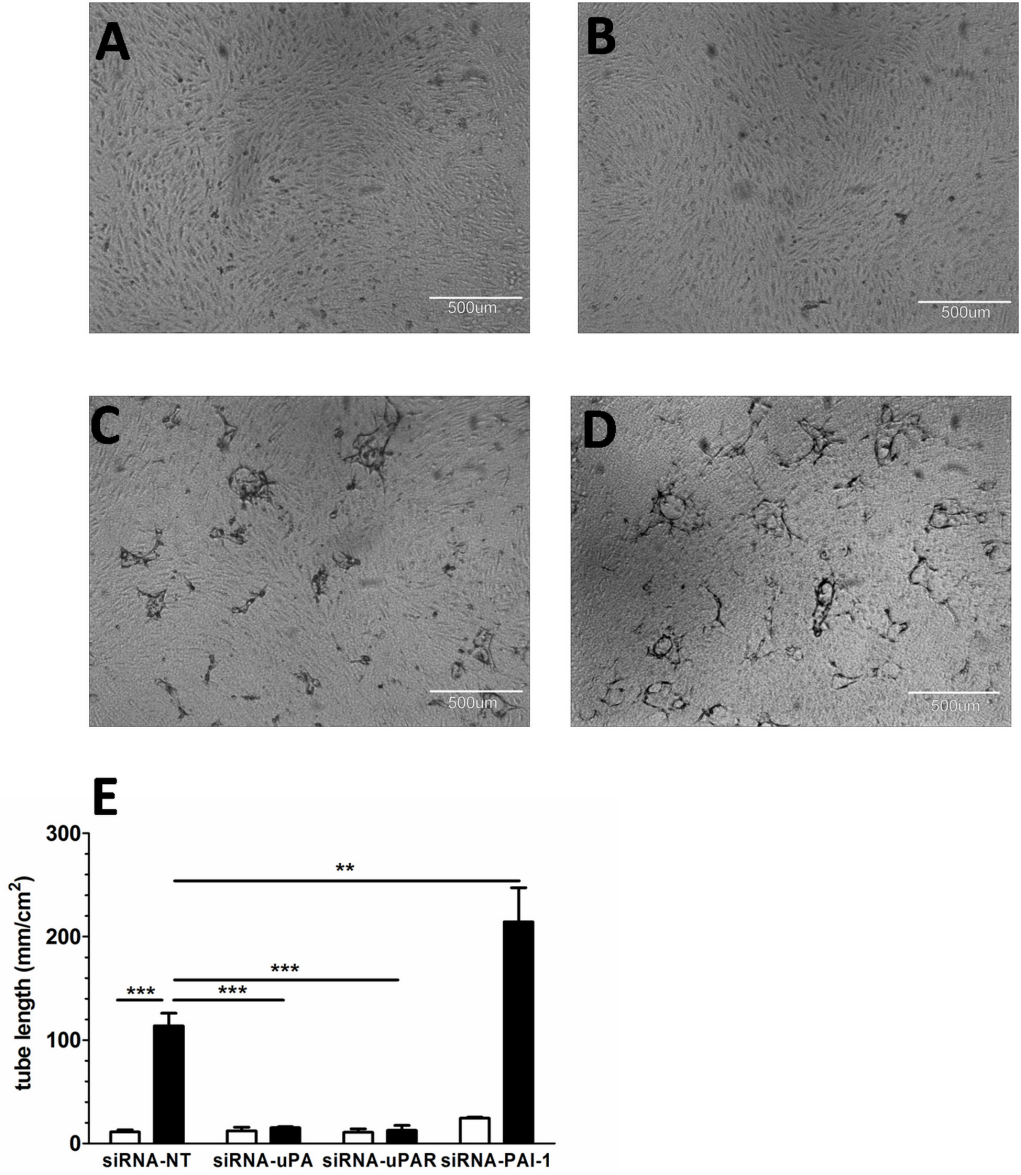
Effect of uPA, uPAR and PAI-1 knockdown on angiogenic response of PB-ECFCs in fibrin matrices. The involvement of uPA/uPAR/PAI-1 system during sprouting in fibrin matrices was assessed in PB-ECFCs obtained from 3 different donors at 18 CPDL. **A** and **B**: diminished angiogenic ability of PB-ECFCs transfected with siRNA-uPA or siRNA-uPAR in fibrin matrices. **C:** sprout formation by cells transfected with non-targeting, control siRNA (si-RNA-NT). **D**: sprout formation by cells transfected with siRNA-PAI-1 (si-RNA-PAi-1). **E:** Comparison of angiogenic response of PB-ECFCs transfected with non-targeting siRNA (siRNA-NT) and siRNA targeting uPA (siRNA-uPA), uPAR (siRNA-uPAR) or siRNA-PAI-1 (si-RNA-PAi-1) expressed as mean ± SEM of mean tube length of tube-like structures of 3 independent experiments of 3 different donors (open bars: unstimulated cells, black bars: cells stimulated with 10ng/mL TNF-α + 10ng/mL FGF-2). Comparison between different groups was performed using two-way ANOVA with Bonferroni post hoc test (p<0.05).

Given the increased expression of the uPA, uPAR and PAI-1 genes during serial propagation of PB-ECFCs in PL, we next assessed their impact on sprout formation in fibrin matrices. The siRNA technology was used to knockdown uPA, uPAR and PAI-1 expression in PB-ECFCs. qRT-PCR confirmed a significant decrease of ~98% in the mRNA levels of uPA, uPAR and PAI-1 when siRNA-uPA, siRNA-uPAR or siRNA-PAI-1 transfected cells were compared with the cells transfected with non-targeting siRNA pool (Fig A in **S4 Fig**). The transfection procedure didn't altered significantly the mRNA levels of uPAR and MMP-14 in siRNA-uPA cells, the uPA and MMP-14 gene expression in siRNA-uPAR cells or uPA, uPAR and MMP-14 in siRNA-PAI-1 cells (Figs B-D in **S4 Fig**). During the course of 3-day stimulation period, the siRNA-NT cells exhibited similar sprouting response as the control, non-transfected cells (Fig E in **S4 Fig**). On the other hand, siRNA-uPA or siRNA-uPAR transfected cells completely failed to form sprouting structures in fibrin matrices (**Fig 7A and 7B**). In contrast, silencing of PAI-1 mRNA induced an increase of sprouting in TNF-α+FGF-2 stimulated ECFCs, while non-targeting siRNA had no effect (**Fig 7C and 7D**). In line with an involvement of the fibrinolytic system, the plasmin inhibitor aprotinin also fully prevented sprout formation (data not shown). These data indicate that sprout formation in fibrin matrices by PB-ECFCs expanded in PL requires pericellular proteolysis involving uPA/uPAR/plasmin activity, which is balanced by PAI-1.

### Induction of activation markers during serial propagation of PB-ECFCs

Progressive upregulation of VEGFR2 in cord blood-derived ECFCs during the expansion process might underlie the increased activation of uPA/uPAR[25]. However, we could not detect a significant upregulation of VEGFR2 mRNA level during long-term expansion of PB-ECFCs in PL-EGM (**S5 Fig**).

**Fig 8:**
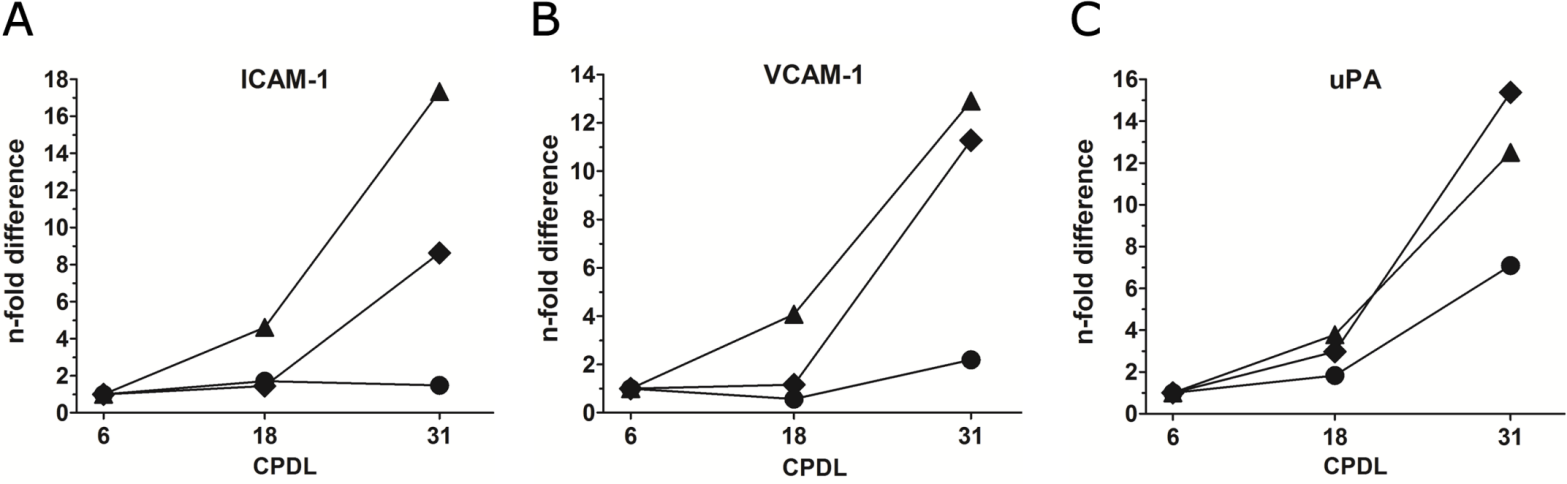
Expression of inflammatory activation-related markers. Quantitative RT-PCR analysis was performed on total cellular mRNA isolated from 3 donors at 6, 18, and 31 CPDL. Gene expression levels of ICAM-1 (**A**), VCAM-1 (**B**), and uPA (**C**) in individual donors PB15 (◆), PB84 (●), and PB224(▲), during long-term expansion. Analysis of uPA expression was performed with the same data set as depicted in Fig 7A. Data are expressed as n-fold difference of expression of same genes in cells at 6 CPDL.

As uPA can be induced by several factors including the inflammatory mediator TNF-α, we subsequently evaluated whether a general inflammatory activation accompanied serial propagation of PB-ECFCs. To that end, we compared the induction of uPA in PB-ECFCs cultures of three different donors with that of VCAM-1, ICAM-1, E-selectin, IL-8, and MCP-1, five factors that can also be induced by TNF-α in endothelial cells. We indeed observed activation of these genes after extensive serial propagation, but the three donors tested differed in the onset of activation as reflected by the five markers, of which VCAM and ICAM-1 are shown in **Fig 8**. Cells at 18 CPDL displayed limited induction of inflammatory markers, but in two donors marked activation was observed at CPDL31. Comparison of the patterns of these five markers with that of the induction of uPA shows a similar pattern for uPA at several time points indicating that the increase of mRNA levels of uPA runs parallel with the other proteins (**Fig 8**).

## Discussion

In the present study we demonstrate an improved method for the isolation of ECFCs from adult peripheral blood. The presented procedure is based on use of human platelet lysate as a serum substitute and yields more colonies per mL collected blood compared to the conventional isolation with FBS. Isolated PB-ECFCs displayed a higher proliferative ability in medium supplemented with platelet lysate than cells expanded in medium with FBS allowing a faster generation of sufficient number of cells for clinical applications. Furthermore, our data showed that an increase of sprouting capacity of PB-ECFCs in fibrin matrices during long-term expansion in platelet lysate is accompanied by an increase at protein and mRNA level of fibrinolytic system. Thus, presented isolation method and further cell expansion in platelet lysate permits optimal large-scale propagation of angiogenic potent PB-ECFCs.

In the present manuscript we demonstrated that the initial yield and expansion of ECFCs from adult peripheral blood by a renewed method using platelet lysate (PL) is stimulated to a larger extent by PL- than by FBS-containing medium. The obtained endothelial cells showed limited telomerase shortening, continued CD34+ expression, and maintenance or increase of sprouting activity in fibrin matrices, which was inhibited by blocking expression of uPA and uPAR. Furthermore, they displayed a limited but gradual increase in expression of inflammation-associated genes in particular after 18 CPDL. Thus the use of platelet lysate provides a humanized condition that enables isolation and large scale propagation of angiogenic potent PB-ECFCs.

### Isolation and characterization of PB-ECFCs in platelet lysate-supplemented medium

The conventional method for the isolation and expansion of peripheral blood-derived ECFCs include supplementation of cell culture medium with 10–20% (v/v) of FBS. However, such procedures yield cells possibly inapt for future clinical application due to the safety concerns related to the use of FBS in preparation of adult stem cells[9]. Isolation of ECFCs in animal serum-free conditions according to the requirements of good manufacture practice has been previously reported in human serum[26–27] and platelet lysate[18]. Data from our lab suggest that isolation of PB-ECFCs in 5% autologous plasma is feasible, yet the limited amounts of donor-derived plasma hampers long-term expansion of ECFCs to numbers sufficient for clinical treatment. It should be noted that—in our attempts to replicate the method with PL proposed by Reinisch et al.[18]—we and others[19] did not succeed in isolating ECFCs from peripheral blood obtained from various individual donors. However, by employing density gradient separation of MNCs from peripheral blood as well as human collagen as coating substrate we were able to isolate ECFCs from adult blood in *in vitro* settings based on the use of platelet lysate. Immunofluorescence phenotyping and functional assays confirmed that PB-ECFCs isolated and cultured in platelet lysate exhibit endothelial lineage markers, and are able to form tube-like structures which is in accordance with previous reports[6]. In addition, these cells are devoid of CD45 and CD14 antigen indicating that PB-ECFCs isolated by the presented procedure do not represent cells from a hematopoietic/monocytic lineage.

### Clonal outgrowth and proliferative capacity of PB-ECFCs in platelet lysate

The number of colonies generated in FBS-EGM and PL-EGM is higher compared to the current widespread procedure for isolation of ECFCs in FBS. In FBS-EGM we obtained two-fold more colonies compared to previous reports[8–28]. Coating with human collagen type I instead rat-tail collagen type I and use of 48-well instead standard 6-well plates may contribute to the difference in number of counted colonies. The presence of heparin in FBS-EGM might also contribute to better clonal outgrowth observed in our study via modulating the activity of heparin-binding sites on VEGF-A[29] and FGF-2[30] which are also present as a supplement in isolation medium. Nevertheless, the abundance of the many growth factors that are released from the platelets, such as VEGF, EGF, HGF, PDGF, and IGF[31] might contribute to enhanced clonal outgrowth of ECFCs in PL compared to FBS and increased proliferation rates. This grants harvest of sufficient number of10^9^-10^11^ cells for clinical use after 15–20 CPDL (for comparison see reference [32]). The cultures of PB-ECFCs at indicated CPDL exhibit a rather stable telomere length and are devoid of significant presence of senescent cells, in accordance with previously reports on a low rate of intrinsic senescence in ECFCs[6,33]. Moreover, the expression of the tip-cell related surface protein CD34 by a significant portion of PB-ECFCs suggests the presence of cells with robust sprouting capacity. Therefore, with respect to the isolation and further expansion of PB-ECFCs in platelet lysate, our improved method offers yield of enough cells with robust angiogenic potency for clinical application within 30 days or less after the blood collection.

### Effect of serial passage on in vitro sprouting ability of PB-ECFCs

Serial passage during expansion may affect the response of cells to the environmental stimuli (e.g. growth factors, hypoxia) that are commonly used to study the physiological process such as angiogenic sprouting[34]. Previous comparison of angiogenic ability of ECFCs expanded in medium containing FBS or PL has unraveled that the serum supplement modulates sprouting ability of these cells[35]. Data from our lab is also in line with that observation since ECFCs formed more sprouts when 5%PL was introduced in our fibrin-based tube-formation assay compared to 10% FBS (S6 Fig). However, it should be noted that Hofbauer et al.[35] used a Matrigel assay, which reflects the rapid reorganization of endothelial cells into a network, while we have used a human fibrin matrix, which reflects the body’s own temporary repair matrix, provide true capillary-like endothelial tubules and is suitable for tissue engineering purposes.

In contrast to our expectation, the data from the sprouting assay suggest that extended cell expansion at 18 and 31 CPDL increases tube formation by FGF-2- and VEGF-A stimulated PB-ECFCs in fibrin matrices as compared to their counterparts at 6 CPDL. Similar findings using the Matrigel angiogenesis assay have also been reported by other investigators[25] who suggested a role for VEGFR2 in VEGF-enhanced sprouting. We found no effect of serial passaging on VEGFR2 mRNA, but we cannot excluded an effect on surface density or turnover of VEGFR2. On the other hand, Basire et al.[36] first reported that the robust angiogenic capacity of ECFCs might be contributed to high intrinsic uPA/uPAR proteolytic ability of these cells. The siRNA data in our study further point to the important role of receptor-bound uPA activity in the formation of tubular structures by PB-ECFCs expanded in PL. This finding is also in line with the previous studies from our group that showed that tube formation by cord blood ECFCs expanded in FBS conditions was inhibited by anti-uPA as well as anti-uPAR IgG antibodies[37]. Furthermore, preceding the enlargement of endothelial cell diameter during long-term propagation an increased expression of uPA has been previously observed[38]. Our data is in the line with this observation since PB-ECFCs at 18 and 31 CPDL displayed an increase of fibrinolytic system at gene and protein level and much higher sprouting ability compared to the cells at 6 CPDL. Interestingly, the amount of PAI-1 antigen markedly exceeds the amount of u-PA antigen produced, as can be observed in the Fig 7. This is comparable with earlier findings in endothelial cells[39]. However, although the amount of PAI-1 does modulate u-PA activity and u-PA-dependent tube formation by ECFC, its effect is minor as compared to the contributions of u-PA and UPAR, as these molecules are rate limiting. In contrast, PAI-1 is produced in excess but only part of it is encountered in an active form[40]. The trend of progressive upregulation of uPA, uPAR and PAI-1 genes runs parallel with a general inflammatory activation of ECFCs since the inflammatory markers such as VCAM, and ICAM-1 become upregulated at mRNA level at a later time point during the serial propagation. From these data we conclude that use of expanded ECFCs at CPDL 15–20 will provide cells with no or limited inflammatory activation.

### Sprouting response of PB-ECFCs under inflammatory condition

Our data revealed that addition of TNF-α augments the basal sprouting induced by FGF-2 or VEGF in PB-ECFCs. Foreskin-derived microvascular endothelial cells (hMVEC) also require the simultaneous presence of TNF-α and FGF-2 or VEGF-A to form tube-like structures in fibrin matrices[21] but application of solely TNF-α to the cells inhibits cell growth and is not sufficient to induce sprouting. However, the PB-ECFCs expanded in platelet lysate were able to form tube-like structures upon stimulation with solely TNF-α at any stage of cellular age when sprouting was assayed. In vivo TNF-α can induce sprouting angiogenesis[41], but mutual interaction between different cell types cannot be excluded. Why TNF-α displays this ability in PB-ECFCs and not in hMVEC is still unclear, but one may hypothesize that PB-ECFCs produce an additional growth factor either constitutively or after induction by TNF-α, which is able to induce sprouting together with TNF-α.

In conclusion, the cell expansion of PB-ECFCs in medium supplemented with platelet lysate permits generation of sufficient number of cells in less than 30 days after the initial blood collection. For optimal use of PB-ECFCs in clinical settings, our data suggest that 15–20 CPDL is the most adequate maturation stage. The cells at this time point retained their proliferative capacity and showed increased sprouting ability in fibrin matrices, were devoid of significant presence of senescence as well as exhibited unaltered expression of the markers associated with inflammatory activation. Thus, the presented isolation method and subsequent cell expansion in platelet lysate supplemented culture medium permits suitable large-scale propagation of angiogenic potent PB-ECFCs.

## Supporting Information

S1 Table List of primers. (DOCX)

S1 Fig Outgrowth of PB-ECFCs at the end of isolation procedure. (A): The average number of ECFCs colonies that appeared in culture at the end of isolation. (B): Distribution of colony outgrowth in FBS (open bars) or PL (black bars) after 10 days, between 10–20 days and at the end of isolation period.
(TIF)

S2 Fig Comparison of expression of progenitor-cell marker CD34 on PB-ECFCs at different maturation stages. A): Flow cytometry data represents mean fluorescence intensity calculated as CD34 antibody fluorescence intensity minus autofluorescence of matched isotype antibody. (B):qRT-PCR validation of mRNA levels of CD34 gene in PB-ECFCs at 18 and 31 CPDL are expressed as a n-fold difference of expression of the same genes in the cells at 6 CPDL.
(TIF)

S3 Fig Tube-forming capacity of PB-ECFCs at different time-points of ex vivo expansion upon stimulation with TNF-α. PB-ECFCs obtained from 3 different donors were serially expanded in medium supplemented with PL and the sprouting ability of cells in fibrin matrices was assessed at 6, 18, and 31 CPDL. Cells at indicated CPDL (white bar 6 CPDL, grey bar 18 CPDL, black bar 31 CPDL) were stimulated with TNF-α (A), TNF-α+FGF-2 (TF, B), TNF-α+VEGF-A (TV, C), and TNF-α+FGF-2+VEGF-A (TFV, D). Results represent the mean ± SEM of mean tube length of tube-like structures of 3 independent experiments each performed at indicated CPDL. Comparison between each CPDLs was performed using one-way ANOVA with Bonferroni post hoc test.(*p < 0.05).
(TIF)

S4 Fig qRT-PCR validation of transfection efficiency. A: Quantitative RT-PCR analysis was performed on total cellular mRNA isolated from PB-ECFCs of three donors transfected with non-targeting siRNA (siRNA-NT) and siRNA targeting uPA (siRNA-uPA), uPAR (siRNA-uPAR) or PAI-1 (si-RNA-PAi-1). Gene expression levels of uPA in siRNA-uPA cells (white bar), uPAR (grey bar) in siRNA-uPAR and PAI-1 (dark grey bar) in siRNA-PAI-1 cells expressed as n-fold difference of expression of the same genes in cells transfected with non-targeting siRNA (black bar). B: Gene expression levels of uPAR and MMP-14 (white bars) in siRNA-uPA cells expressed as n-fold difference of expression of the same genes in cells transfected with non-targeting siRNA (black bar). C: Gene expression levels of uPA and MMP-14 (white bars) in siRNA-uPAR cells expressed as n-fold difference of expression of the same genes in cells transfected with non-targeting siRNA (black bar). D: Gene expression levels of uPA, uPAR and MMP-14 (white bars) in siRNA-PAI-1 cells expressed as n-fold difference of expression of the same genes in cells transfected with non-targeting siRNA (black bar). E: Comparison of angiogenic response of PB-ECFCs transfected with non-targeting siRNA (siRNA-NT) to control, untransfected cells expressed as mean ± SEM of mean tube length of tube-like structures of 3 independent experiments of 3 different donors (open bars: unstimulated cells, black bars: cells stimulated with 10ng/mL TNF-α + 10ng/mL FGF-2, gray bars: cells stimulated with 10ng/mL TNF-α + 10ng/mL FGF-2 + 100U/mL aprotinin).
(TIF)

S5 Fig Gene expression levels of VEGFR2 in PB-ECFCs at different maturation stages. Quantitative RT-PCR analysis was performed on total cellular mRNA isolated from PB-ECFCs of three donors at different CPDL (open bar 6 CPDL, grey bar 18 CPDL, black bar 31 CPDL). Data are expressed as n-fold difference of expression ofsame genes in cells at 6CPDL. One-way ANOVA with Bonferroni post hoc test (p<0.05).
(TIF)

S6 Fig Tube-forming capacity of PB-ECFCs in presence of FBS or PL. The tube-forming effect of FBS or PL on PB-ECFC isolated and expanded in PL was investigated after stimulation with 10ng/mL VEGF-A prepared in M199+10%FBS+10U/mL heparin or M199 +5%PL+10U/mL heparin. Results represent the mean ± SEM of mean tube length of tube-like structures of 3 independent experiments. Comparison between each CPDLs was performed using one-way ANOVA with Bonferroni post hoc test.(*p < 0.05).
(TIF)
