# Supplementary material for: Long-Term Expansion in Platelet Lysate Increases Growth of Peripheral Blood-Derived Endothelial-Colony Forming Cells and Their Growth Factor-Induced Sprouting Capacity
Source: PLoS One. 2015 Jun 15;10(6):e0129935. doi: 10.1371/journal.pone.0129935 (PMC4468160; doi:10.1371/journal.pone.0129935)
Supplement: S1 Table — (DOCX) [file pone.0129935.s001.docx]

**Supplementary table 1, Tasev et al.**

| **Gene** | **Forward primer** | **Reverse primer** |
| --- | --- | --- |
| GAPDH^1^ | GGTCTCCTCTGACTTCAACA | AGCCAAATTCGTTGTCATAC |
| CD34^2^ | ACCACTAGCACTAGCCTTGC | CCTTCTTAAACTCCGCACAGC |
| uPA^3^ | ACTACTACGGCTCTGAAGTCACCA | GAAGTGTGAGACTCTCGTGTAGAC |
| uPAR^4^ | CATGCAGTGTAAGACCAACGGGGA | AATAGGTGACAGCCCGGCCAGAGT |
| tPA^3^ | CCAGATCGAGACTCAAAGCC | GACCCATTCCCAAAGTAGCA |
| PAI-1^3^ | GCACAACCCCACAGGAAC | TGCTTCAAACTTCTCTCCCAG |
| VEGFR-2^5^ | TGGGAACCGGAACCTCACTATC | GTCTTTTCCTGGGCACCTTCTATT |
| ICAM-1 | CTCAAAAGTCATCCTGCCCCG | GGCAGCGTAGGGTAAGGTTCT |
| VCAM-1^6^ | ACAAAGGCAGAGTACGCAAACA | GGCTGACCAAGACGGTTGTATC |
| MMP-14^7^ | GCAGAAGTTTTACGGCTTGCAA | CCTTCGAACATTGGCCTTGAT |
| MMP-2^8^ | CAACTACAACTTCTTCCCTCGCA | GGTCACATCGCTCCAGACTTG |

1. Brooks, S. P., Trueman, R. C. & Dunnett, S. B. Striatal lesions in the mouse disrupt acquisition and retention, but not implicit learning, in the SILT procedural motor learning task. *Brain Res.* **1185,** 179–188 (2007).

2. Brunet De La Grange, P. *et al.* Oxygen concentration influences mRNA processing and expression of the cd34 gene. *J. Cell. Biochem.* **97,** 135–144 (2006).

3. Houard, X. *et al.* Topology of the fibrinolytic system within the mural thrombus of human abdominal aortic aneurysms. *J. Pathol.* **212,** 20–28 (2007).

4. Li, Y. & Sarkar, F. H. Down-regulation of invasion and angiogenesis-related genes identified by cDNA microarray analysis of PC3 prostate cancer cells treated with genistein. *Cancer Lett.* **186,** 157–164 (2002).

5. Smadja, D. M. *et al.* PAR-1 activation on human late endothelial progenitor cells enhances angiogenesis in vitro with upregulation of the SDF-1/CXCR4 system. *Arterioscler. Thromb. Vasc. Biol.* **25,** 2321–2327 (2005).

6. Wolf, S. C. *et al.* Influence of Nebivolol and Metoprolol on Inflammatory Mediators in Human Coronary Endothelial or Smooth Muscle Cells. Effects on Neointima Formation After Balloon Denudation in Carotid Arteries of Rats Treated with Nebivolol. *Cell. Physiol. Biochem.* **19,** 129–136 (2007).

7. Munoz-Najar, U. M., Neurath, K. M., Vumbaca, F. & Claffey, K. P. Hypoxia stimulates breast carcinoma cell invasion through MT1-MMP and MMP-2 activation. *Oncogene* **25,** 2379–2392 (2005).

8. Mu, H. *et al.* Adipokine resistin promotes in vitro angiogenesis of human endothelial cells. *Cardiovasc. Res.* **70,** 146–157 (2006).
