# Supplementary figures and images for: Long-Term Expansion in Platelet Lysate Increases Growth of Peripheral Blood-Derived Endothelial-Colony Forming Cells and Their Growth Factor-Induced Sprouting Capacity
Source: PLoS One. 2015 Jun 15;10(6):e0129935. doi: 10.1371/journal.pone.0129935 (PMC4468160; doi:10.1371/journal.pone.0129935)

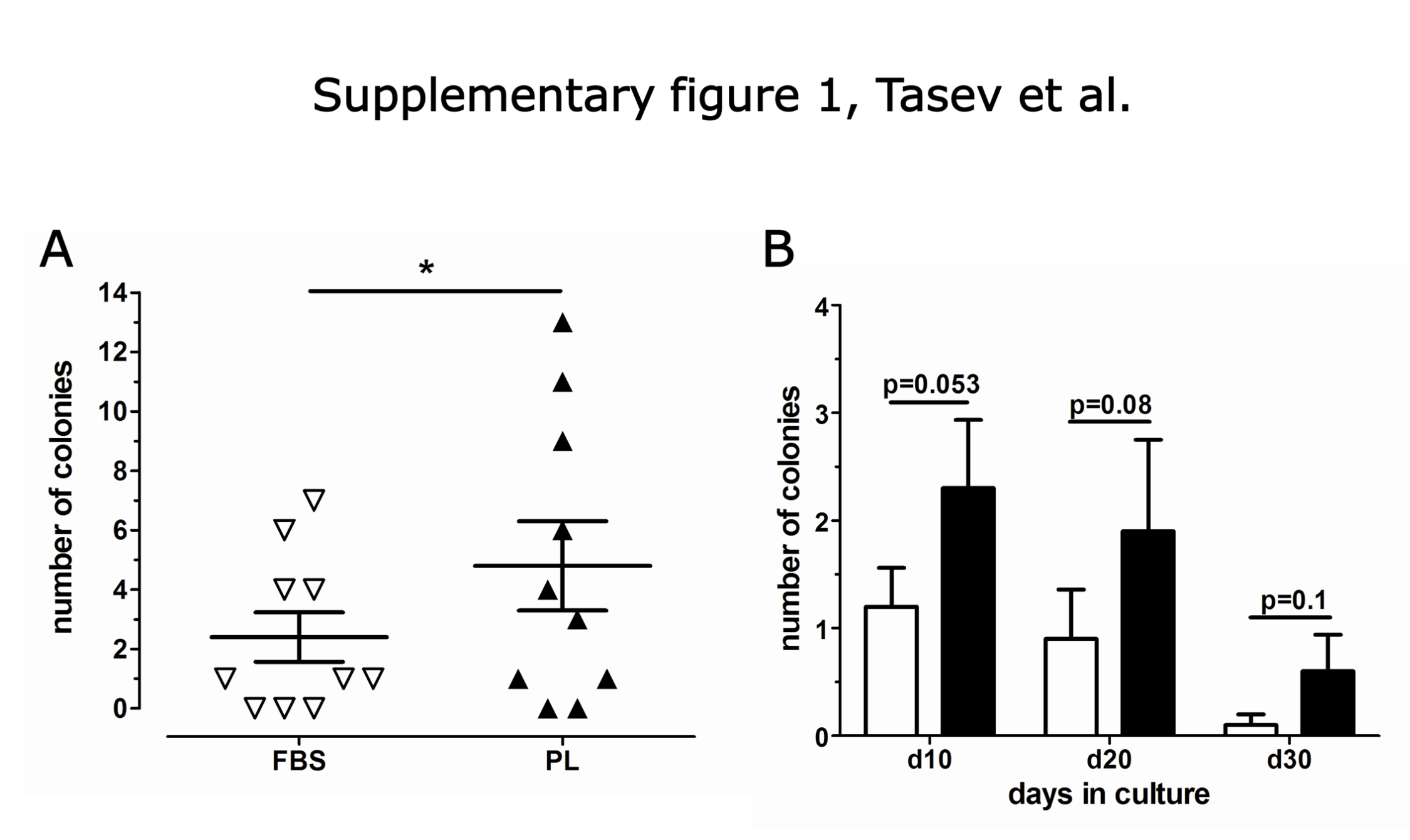

Supplement: S1 Fig — (A): The average number of ECFCs colonies that appeared in culture at the end of isolation. (B): Distribution of colony outgrowth in FBS (open bars) or PL (black bars) after 10 days, between 10–20 days and at the end of isolation period. (TIF) [file pone.0129935.s002.tif]

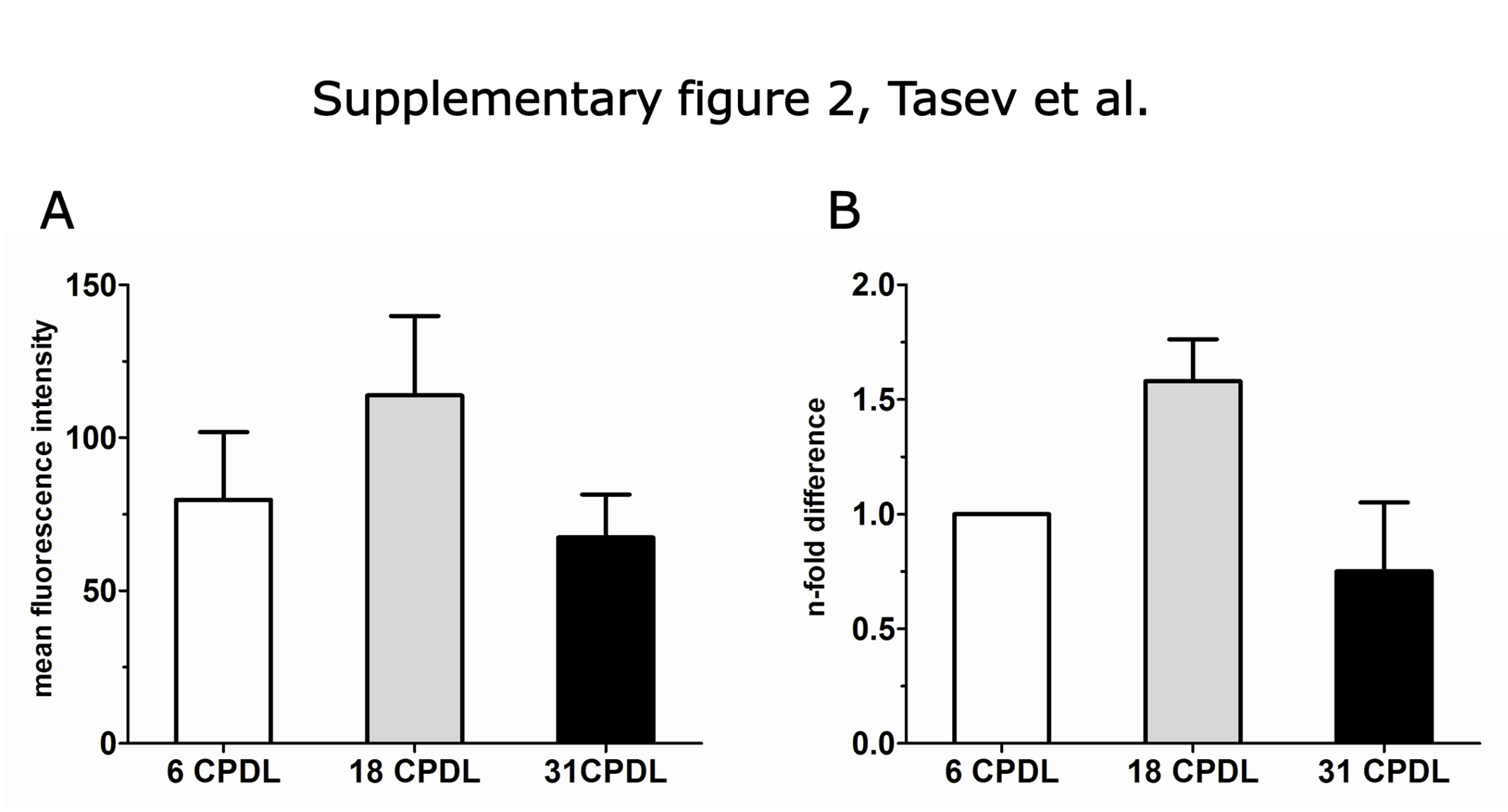

Supplement: S2 Fig — A): Flow cytometry data represents mean fluorescence intensity calculated as CD34 antibody fluorescence intensity minus autofluorescence of matched isotype antibody. (B):qRT-PCR validation of mRNA levels of CD34 gene in PB-ECFCs at 18 and 31 CPDL are expressed as a n-fold difference of expression of the same genes in the cells at 6 CPDL. (TIF) [file pone.0129935.s003.tif]

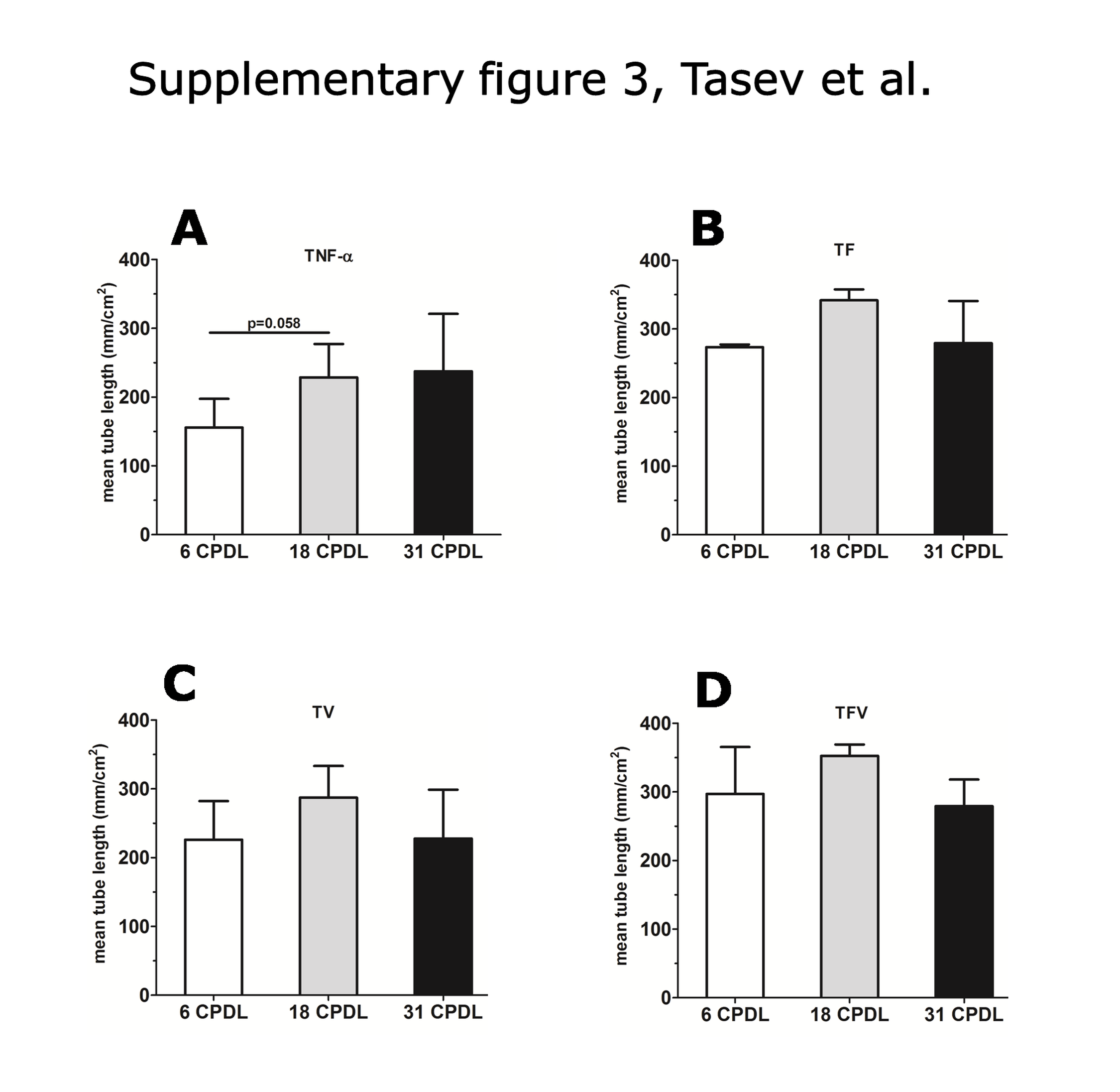

Supplement: S3 Fig — PB-ECFCs obtained from 3 different donors were serially expanded in medium supplemented with PL and the sprouting ability of cells in fibrin matrices was assessed at 6, 18, and 31 CPDL. Cells at indicated CPDL (white bar 6 CPDL, grey bar 18 CPDL, black bar 31 CPDL) were stimulated with TNF-α (A), TNF-α+FGF-2 (TF, B), TNF-α+VEGF-A (TV, C), and TNF-α+FGF-2+VEGF-A (TFV, D). Results represent the mean ± SEM of mean tube length of tube-like structures of 3 independent experiments each performed at indicated CPDL. Comparison between each CPDLs was performed using one-way ANOVA with Bonferroni post hoc test.(*p < 0.05). (TIF) [file pone.0129935.s004.tif]

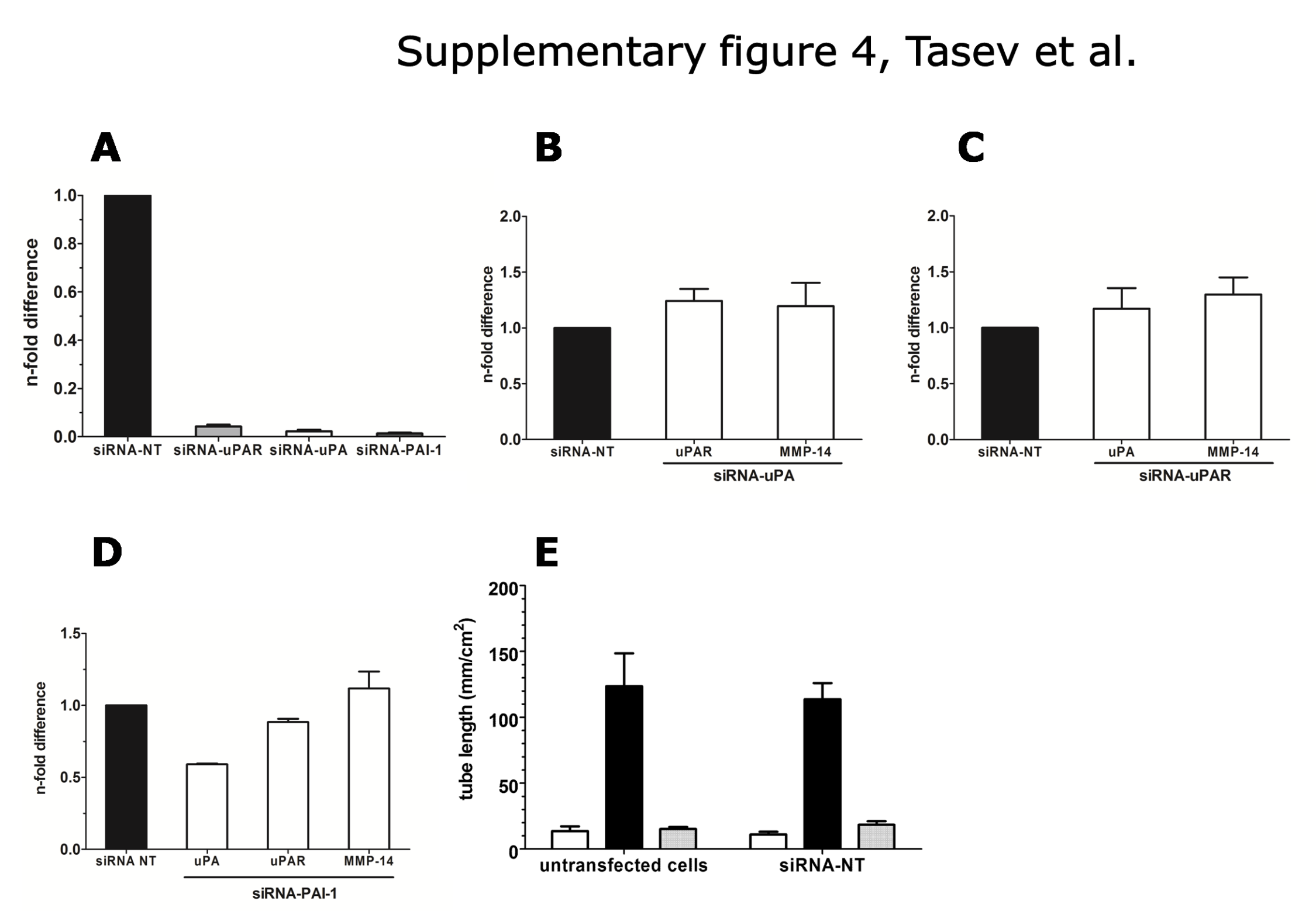

Supplement: S4 Fig — A: Quantitative RT-PCR analysis was performed on total cellular mRNA isolated from PB-ECFCs of three donors transfected with non-targeting siRNA (siRNA-NT) and siRNA targeting uPA (siRNA-uPA), uPAR (siRNA-uPAR) or PAI-1 (si-RNA-PAi-1). Gene expression levels of uPA in siRNA-uPA cells (white bar), uPAR (grey bar) in siRNA-uPAR and PAI-1 (dark grey bar) in siRNA-PAI-1 cells expressed as n-fold difference of expression of the same genes in cells transfected with non-targeting siRNA (black bar). B: Gene expression levels of uPAR and MMP-14 (white bars) in siRNA-uPA cells expressed as n-fold difference of expression of the same genes in cells transfected with non-targeting siRNA (black bar). C: Gene expression levels of uPA and MMP-14 (white bars) in siRNA-uPAR cells expressed as n-fold difference of expression of the same genes in cells transfected with non-targeting siRNA (black bar). D: Gene expression levels of uPA, uPAR and MMP-14 (white bars) in siRNA-PAI-1 cells expressed as n-fold difference of expression of the same genes in cells transfected with non-targeting siRNA (black bar). E: Comparison of angiogenic response of PB-ECFCs transfected with non-targeting siRNA (siRNA-NT) to control, untransfected cells expressed as mean ± SEM of mean tube length of tube-like structures of 3 independent experiments of 3 different donors (open bars: unstimulated cells, black bars: cells stimulated with 10ng/mL TNF-α + 10ng/mL FGF-2, gray bars: cells stimulated with 10ng/mL TNF-α + 10ng/mL FGF-2 + 100U/mL aprotinin). (TIF) [file pone.0129935.s005.tif]

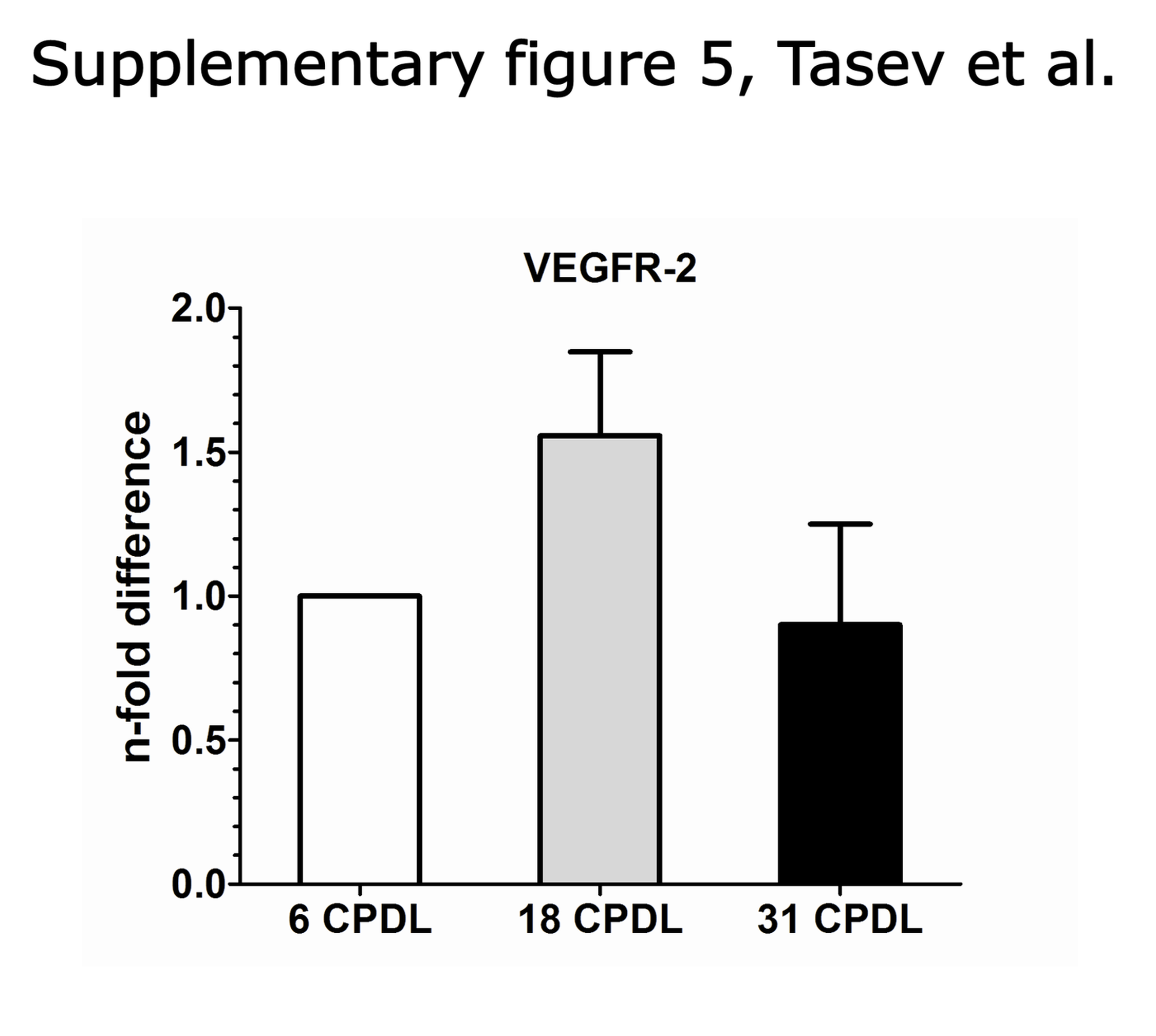

Supplement: S5 Fig — Quantitative RT-PCR analysis was performed on total cellular mRNA isolated from PB-ECFCs of three donors at different CPDL (open bar 6 CPDL, grey bar 18 CPDL, black bar 31 CPDL). Data are expressed as n-fold difference of expression ofsame genes in cells at 6CPDL. One-way ANOVA with Bonferroni post hoc test (p<0.05). (TIF) [file pone.0129935.s006.tif]

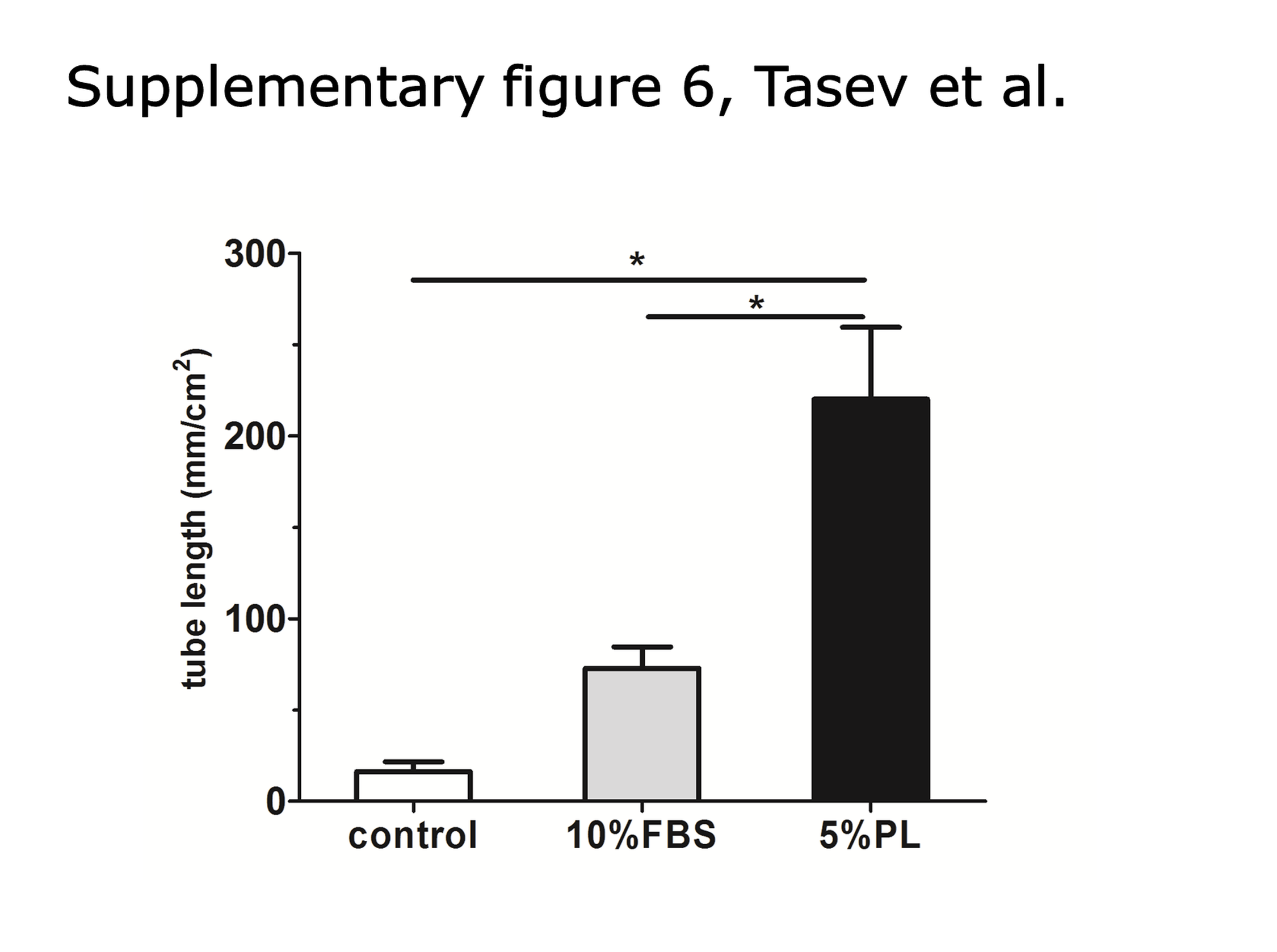

Supplement: S6 Fig — The tube-forming effect of FBS or PL on PB-ECFC isolated and expanded in PL was investigated after stimulation with 10ng/mL VEGF-A prepared in M199+10%FBS+10U/mL heparin or M199 +5%PL+10U/mL heparin. Results represent the mean ± SEM of mean tube length of tube-like structures of 3 independent experiments. Comparison between each CPDLs was performed using one-way ANOVA with Bonferroni post hoc test.(*p < 0.05). (TIF) [file pone.0129935.s007.tif]
